# A reliable Epstein-Barr Virus classification based on phylogenomic and population analyses

**DOI:** 10.1038/s41598-019-45986-3

**Published:** 2019-07-08

**Authors:** Louise Zanella, Ismael Riquelme, Kurt Buchegger, Michel Abanto, Carmen Ili, Priscilla Brebi

**Affiliations:** 1 Laboratory of Integrative Biology (LIBi), Universidad de La Frontera, Temuco, Chile; 2 Center for Excellence in Translational Medicine (CEMT), Universidad de La Frontera, Temuco, Chile; 3 Scientific and Technological Bioresource Nucleus (BIOREN), Universidad de La Frontera, Temuco, Chile; 4 Instituto de Ciencias Biomédicas, Facultad de Ciencias de la Salud, Universidad Autónoma de Chile, Temuco, Chile

**Keywords:** Phylogeny, Phylogenetics

## Abstract

The Epstein-Barr virus (EBV) infects more than 90% of the human population, playing a key role in the origin and progression of malignant and non-malignant diseases. Many attempts have been made to classify EBV according to clinical or epidemiological information; however, these classifications show frequent incongruences. For instance, they use a small subset of genes for sorting strains but fail to consider the enormous genomic variability and abundant recombinant regions present in the EBV genome. These could lead to diversity overestimation, alter the tree topology and misinterpret viral types when classified, therefore, a reliable EBV phylogenetic classification is needed to minimize recombination signals. Recombination events occur 2.5-times more often than mutation events, suggesting that recombination has a much stronger impact than mutation in EBV genomic diversity, detected within common ancestral node positions. The Hierarchical Bayesian Analysis of Population Structure (hierBAPS) resulted in the differentiation of 12 EBV populations showed seven monophyletic and five paraphyletic. The populations identified were related to geographic location, of which three populations (EBV-p1/Asia/GC, EBV-p2/Asia II/Tumors and EBV-p4/China/NPC) were related to tumor development. Therefore, we proposed a new consistent and non-simplistic EBV classification, beneficial in minimizing the recombination signal in the phylogeny reconstruction, investigating geography relationship and even infer associations to human diseases. These EBV classifications could also be useful in developing diagnostic applications or defining which strains need epidemiological surveillance.

## Introduction

The Epstein-Barr virus (EBV) is one of the world’s most disseminated viruses^1^, and has been associated with nonmalignant diseases such as infectious mononucleosis (IM)^2^ and post-transplant lymphoproliferative disease (PTLD)^3^, together with several cancers including Burkitt’s lymphoma (BL)^4^, gastric cancer (GC)^5^, Hodgkin’s lymphoma (HL)^6^, lung carcinoma (LC)^7^, nasopharyngeal carcinoma (NPC)^8^. EBV is a member of the *Herpesviridae* family, more specifically, belonging to the *Gammaherpesvirinae* subfamily and renamed as *Human herpesvirus 4* (HHV4)^9^. Its complex genome is composed of a linear double-stranded DNA of ∼170 kb in length, including around 80 possible coding regions (CDS)^10^, a series of repeat regions (terminal direct repeats, TRs, internal repeat sequences, IRs), and large indel regions, found in some cancer biopsies and cancer cell-line cultures^11–13^.

Many attempts have been made to classify EBV according to clinical or epidemiological information. Initially, EBV was classified into two types: EBV type 1 (EBV-1) and EBV type 2 (EBV-2) (type A and type B), based on alleles at the EBNA2 and EBNA-3A, -3B and -3C genes^14^. One of the clinical differences between them is that type 1 can convert B cells into lymphoblastoid cell lines (LCL) more efficiently than type 2^15,16^. The geographical distribution of EBV types shows that type 1 is the most prevalent in the world, predominantly in Europe, Asia and North and South America. Instead, type 2 is more frequent in Alaska, Papua New Guinea and Central Africa^17^, with far higher frequency in countries like Kenya^18^. In fact, dual infections with these EBV types have also been reported in these locations^18,19^, including intertypic recombinants with type 1 EBNA2 and type 2 EBNA3s^20–22^.

Other studies tried to classify this virus by geographic location sampling or according to single gene or small subset of EBV gene polymorphisms involved in certain pathologies^23,24^. Some classifications are based on variations of LMP-1 and EBNA-1 genes, for instance, one study analyzed part of the LMP-1 gene sequences to primarily establish 7 EBV strains^25,26^. However, after studying the complete LMP1 gene sequence, this classification was redefined into 6 EBV strains: Alaskan (AL), China1, China2, B95.8 (Med+), Med− and Argentine, indicating the impact of partial sequences to classify an organism^26,27^. Moreover, mixed LMP1 haplotypes were also reported^27^. Similar analysis have been performed to study the variations of EBNA-1 gene^28^, however, the association between pathological conditions and these subtypes is still controversial^29–31^.

Although the genotyping scheme of type 1/type2 classification is widely accepted, Santpere *et al*.^13^ have commented of abundant recombination events existing within EBV genome, because they could lead to classification errors. In fact, McGeoch *et al*.^32^ hypothesized that EBV type 2 strain results from recombination events involving EBNA2 and EBNA3s genes, occurring between EBV type 1 strain and closely-related lymphocryptovirus, or the positive selection of some genes variants. Unfortunately, few studies have assessed the effect of the recombination on classification and evolutionary history of EBV^13,33^. Therefore, EBV classification should not be limited to the study of a few genes or partial viral sequences, but, on the contrary, it is necessary to reanalyze the entire EBV genome with special attention to recombination events.

Here, a comprehensive landscape of EBV recombinant regions is provided to establish the impact on viral phylogenetic reconstruction, proposing a new classification of EBV dealing with effects of recombination based on population structure analysis combined with phylogeny.

## Methods

### Sequence datasets, alignments and phylogenetic inferences

A final dataset of 188 EBV genomes based on samples between 1970 and 2017, including some sequences without collection date (genome list in the Supplementary material, Table S1) were considered, and using NCBI database, last accessed on June 26, 2018 (https://www.ncbi.nlm.nih.gov/). This dataset included similar size genomes with the minimum nonspecific bases (Ns). However, due to lack of information and contradictory origin records of some virus strains, all selected genomes were treated individually albeit some of them are replicates of the same virus (e.g. Mak1 and Makau) or derivative strains (e.g. PH3R1 and Jijoye), reported by Ba Abdullah *et al*.^34^. Next, multiple sequence alignment was performed using MAFFT (http://mafft.cbrc.jp/alignment/software/)^35^, the final alignment being reported in the supplementary material. Literature mining was used to identify nine critical genes during EBV infection and previously used in phylogenetic analyses. The following genes were involved in **latent cycle**: (i) EBNA1, EBNA2, EBNA3A (exon 1-2), EBNA3B (exon 1-2), EBNA3C (exon 1-2), LMP1 (exon 1-3) and LMP2 (coding isoform A, exon 1-8); and in **lytic cycle**: (ii) BLLF1 (coding isoform of gp350) and BZLF1 (exon 1-3).

Afterwards, specific alignments for these selected coding regions (CDS), excluding introns, were individually created based on initial genome alignment. The poorly aligned sites and divergent regions of each dataset were separately extracted by Gblocks^36^, such as sequences containing likely errors (GD2 strain isolate) preventing the generation of artificial genomic diversity, whilst avoiding assigning a greater phylogenetic weight to certain strains. Subsequently, alignments were manually inspected using BioEdit 7.2.5^37^ prior to construction of phylogenetic trees. The best-fit nucleotide substitution model was calculated using jModelTest2^38^, based on the Akaike information criterion (AIC) (Supplementary material, Table S2).

Identification of EBV types by maximum-likelihood (ML) phylogenetic inferences of the EBV genome and CDS alignments were determined using PhyML 3.0 (http://www.atgc-montpellier.fr/phyml/) with 1000 bootstrap value. Figtree 1.4.3 software (http://tree.bio.ed.ac.uk/software/figtree/) was used to visualize the ML trees, and results were treated with iTOL^39^. Topologies of ML trees were evaluated regarding the sufficient phylogenetic signal to discriminate between EBV type 1 and 2 (traditional classification). The sequences of B95-8 Raji (a B95-8/Raji hybrid), Akata, Mutu, C666-1, M81, GD1 and GD2 were used as EBV-1 references, while sequences of AG876, Jijoye, and Wewak1 were used as EBV-2 references (accession numbers shown in the Supplementary material, Table S1)^40^.

### Recombination analysis

After elimination of poorly aligned sites, genomes were re-examined to ensure that recombinant regions were not present. For this purpose, phylogenetic methods (Bootscan^41^ and RDP^42^); and nucleotide substitution methods (Chimaera^43^, GENECONV^44^, MaxChi^44^, SisScan^45^, and 3Seq^46^) were implemented in the RDP4 software package^46^ with default parameters (Supplementary material, Tables S3 and S4). Potential putative recombinant events were considered significant when all six algorithms had the threshold p-value 0.05, using the Bonferroni correction.

Considering that most genomes had putative recombinant portions, and the elimination of such genomes would drastically decrease the number of sequences analyzed, a novel approach was used to identify recombination signals through ClonalFrameML^47^ and Gubbins^48^. ClonalFrameML was used again to mask recombinant portions of the genomes, replacing these recombinant regions with Ns to perform further phylogenetic analyses. These software packages identify regions containing elevated densities of base substitution, as a predictor of potential recombinant regions. The ClonalFrameML (i) identifies and (ii) masks the putative recombinant regions (Supplementary material, Table S5), (iii) estimates the ratios of recombination events relative to point mutations (rho/theta), and (iv) calculates the rates of recombination and mutation (r/m) (Supplementary material, Table S6) To confirm the results obtained by ClonalFrameML and explore the impact of recombination on each specific clade and isolate, Gubbins^48^ software was used. Then, masked alignment of ClonalFrameML was used to generate a maximum likelihood tree with IQ-TREE^49^. To visualize and compare the trees, iTOL and Phytools in the R package^50^ were used, respectively.

### Identification of population groups

A Bayesian analysis (hierBAPS)^51^ was used to determine the population structure of EBV genomes with filtered recombination sites (masked alignment). Clustering was performed with four hierarchy levels and, in order to identify optimal clustering, 10 independent interactions were run using a prior upper boundary of 20 clusters set for each alignment. For the masked alignment, the estimated clustering mode had 12 clusters at level 2 of the hierarchy.

## Results

### Inconsistencies in tree topologies

Previous reports mostly tried to reconstruct the EBV phylogenetic relationship using a low sample number and applying strategies based on one gene, without considering inconsistencies found when more genes are used. To address this, we explored the EBV phylogenetic relationships influenced by genomic regions and recombination events through ML reconstructions from whole genome and nine CDS: BLLF1 (isoform gp350) envelope glycoprotein responsible to viral attachment to host cells^52^; BZLF1, a transcriptional activator mediating the switch between latent and lytic cycle of EBV infection^53^, EBNAs (EBNA–1, –2 and –3s) latency genes; and LMPs (LMP1 and LMP2A isoforms) encoding latent membrane protein inducing transformation of the host cell^26^.

**Figure 1:**
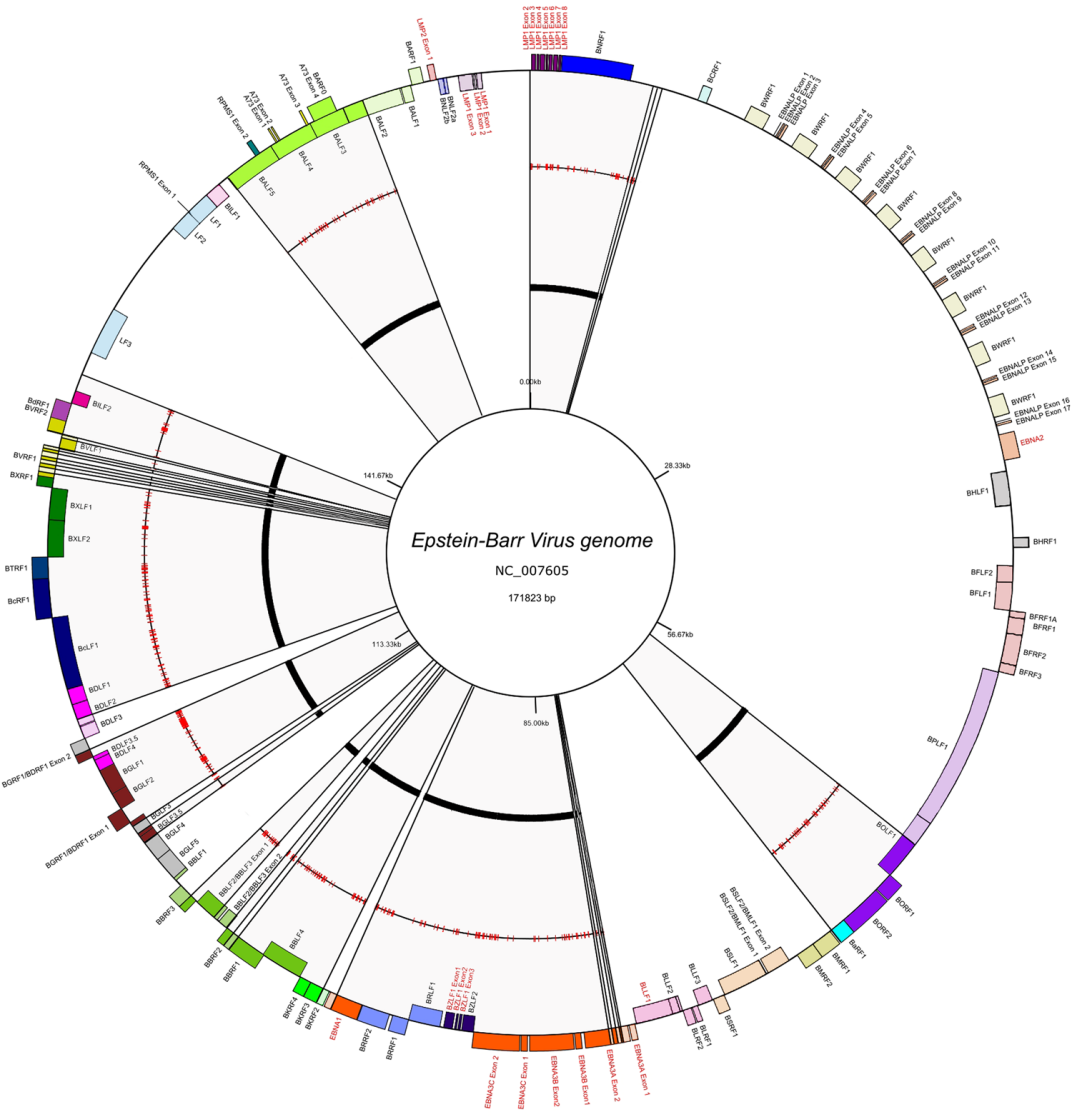
Map of EBV genome. Circles from inner to outer display the genome reference NC_007605 coordinates (innermost circle), the second circle indicates remaining regions after removing poorly aligned sites. Third circle highlights the recombination sites in red. The outermost circle displays a gene map, where genes shown outside of circle are transcribed clockwise, whereas those shown inside are transcribed counterclockwise. The genes used in ML analyses are highlighted by red labels. This figure was performed by using GenomeVx (http://wolfe.ucd.ie/GenomeVx/).

Poorly aligned positions, with excessive gap alignment and considerable divergent regions, were removed by ClonalFrameML, individually in both partial (CDS) and whole genome alignments. For whole genome alignments, some genes were eliminated by considering uneven nucleotide content. The exclusion of these poorly aligned sites reduced the genome size from ~175,000 base pairs (bp) to ~85,000 bp, with a final length representing ~48% of the original sequence. A representation of EBV genome including the nine CDS used and the remaining sequences (after above filtration), are depicted in Fig. 1.

**Figure 2:**
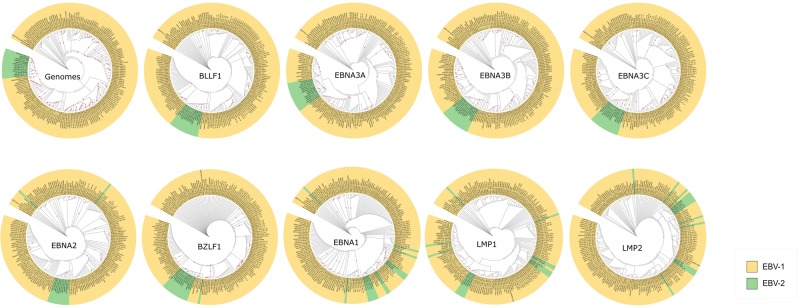
Comparison of phylogenetic trees according to EBV-1 and EBV-2 classification. Phylogenetic ML trees based on sequences of entire genome (~85000pb), BLLF1 (2228pb), EBNA3A (2659pb), EBNA3B (2286pb), EBNA3C (2577pb), EBNA2 (1095pb), BZLF1 (801pb), EBNA1 (1019pb), LMP1 (1088pb) and LMP2 (1418pb). In green the isolates classified as EBV-2 and in yellow the isolates classified as EBV-1. The five trees on top could not be used to distinguish between EBV type 1 and type 2. The red triangles indicate bootstrap values above 80%.

Then, phylogenetic analyses were conducted on whole EBV genome and specific CDS regions: BLLF1, BZLF1, EBNAs, and, LMPs genes, excluding intronic regions. Tree topologies among different genes were similar; however, most of well-supported nodes were found in the whole genome tree (Fig. 2, red triangles on the nodes), which had more than 80% bootstrap support. Conversely, in the case of CDS trees, the EBNA2 had the lowest number of well-supported nodes (only 23), which could be explained by the conserved gene nature having key roles in EBV infection.

As geographic association of EBV is a commonly explored topic, we identified major phylogenetic clades using CDS and whole genome trees according to their source. The Australia-UK clade containing isolates from Australia and the United Kingdom (sLCL-IM1.05-IM, sLCL-IM1.02, sLCL-IS1.13, HL04, HL05 and HL09), and two clades containing exclusively sequences from Kenya: Kenya I clade (sLCL-1.02, sLCL-BL1.03, sLCL-1.04, sLCL-1.06, sLCL-1.07, sLCL-1.08, sLCL-1.10, sLCL-1.11, sLCL-BL1.20 and Mutu) and Kenya II clade (Makau and its resequenced Mak1duplicate) were present in all trees. However, in EBNA3C, the Kenya I clade showed one additional isolate: sLCL1.17, probably a recombinant isolate; in fact, this gene showed a recombination rate above genome average^54^. These three clades (Australia-UK, Kenya I and Kenya II) showed a high support (above 89%), but the bootstrapping value with EBNA2 tree was low (61%); therefore, it does not have enough statistical power to distinguish these clades. Moreover, EBNA2 has shown remarkable conserved regions^55^, possibly related with its important role as master transcription regulator.

EBV is commonly classified into two genotypes: type 1 and type 2 based on the EBNA2, EBNA3s genes; type 1 is represented by reference genomes B95.8 (B95-8/Raji hybrid), Akata, Mutu, C666-1, M81, GD1 and GD2; and type 2 by AG876, Jijoye, and Wewak1^40^. Therefore, critical analysis of the phylogenetic signal of each dataset was carried out separating the sequences into type 1 and type 2 genotypes of the traditional classification. In this regard, phylogenetic reconstruction established that type 2 reference isolates (AG876, Jijoye, and Wewak1) clustered with another 11 isolates: P3HR1 (the derivative sequence from Jijoye^56^), Cheptages, BL36 (intertypic recombinant^22^), sLCL-2.14, sLCL-2.16, sLCL1.18, sLCL2.21, sLCL-IS2.01, sLCL-2.15, sLCL-2.22 and Akata-GC1. Type 2 cluster contained a small number of sampled isolates compared to type 1. The total of 14 isolates were recovered with high phylogenetic confidence (100% bootstrap value), using EBNA3s genes and genome datasets to define them, as belonging to type 2 genotype.

Phylogenetic reconstruction using the whole genome, along with BLLF1 and EBNA3s genes could segregate EBV isolates into traditional type 1 and type 2 classification (Fig. 2, on the top). Conversely, phylogeny of BZLF1, EBNA1, LMP1 and LMP2 genes were not consistent with this traditional classification; or similar to results previously reported with EBNA1 and LMPs genes^22,27^.

EBNA2, the most used gene in sorting EBV according to traditional classification^57^, can group most isolates belonging to type 2 cluster, but not all of them (Fig. 2, bottom). In fact, of 14 isolates identified as belonging to EBV type 2 genotype, only 10 were identified as type 2 according to EBNA2 gene: AG876, Jijoye, Wewak, sLCL-2.14, sLCL-2.16, sLCL2.21, sLCL-IS2.01, sLCL-2.15, sLCL-2.22 and Akata-GC1 (supported by 99% of bootstrap value). From the remaining four isolates, two were EBNA2 deficient (Cheptages and P3HR1), similarly to the Daudi isolate belonging to EBV type 1, also lacking EBNA2 gene, as previously reported^11,56,58^. The other two remaining isolates (sLCL1.18 and BL36), cluster within the EBV-2 genotype, according to the whole genome and BLLF1, and EBNA3s genes. The BL36 isolate was previously characterized as the intertypic recombination resulting from a superinfection produced by a type 2 BL and B95-8 virus (EBV1 prototype), suggesting that BL36 is a laboratory artefact and does not represent a natural circulating strain. Conversely, the sLCL1.18 may represent a natural intertypic recombinant^11^.

As demonstrated above, EBNA2 and EBNA3s genes had sufficient phylogenetic signal to discriminate between EBV-1 and EBV-2, confirming the existence of isolates classified as EBNA2 type 1 and EBNA3s type 2. These results led us to study the effect of recombination on both the phylogenetic resolution and the EBV classification.

### Identification of putative recombination

Recombination is an evolutionary process that hinders most to reach a consensus to classify EBV isolates^13,30^. Several inconsistencies have been detected in sorting some EBV isolates (e.g. sLCL-1.18, BL36 and sLCL1.17) within traditional classification. For instance, the BL36 and sLCL-1.18 isolates were previously classified as intertypic recombinant genotype EBNA2 type 1/EBNA3s type 2^22^. To solve these discrepancies, we analyzed putative recombinant regions in the whole genome and BLLF1, EBNA3s genes, which apparently recovered a traditional classification. The first analysis detected the presence of recombination events in the whole genome and EBNA3s genes. Most analyzed isolates showed putative recombination portions in the genome, resulting from 25 identified putative recombination events, being statistically significant: with one in EBNA3A, one in EBNA 3B, and two in EBNA3C (all with p < 0.01 defined by RDP4, see Supplementary material, Table S4). Considering the whole genome and the four CDS analyzed (BLLF1 and EBNAs), the only gene with no evidence of recombination was BLLF1, and the rest of genes (EBNA3s) and the genome shown recombination signals.

Although the number of recombination events is significant within the EBV genome, and lesser in some CDS, the real contribution of these recombination in the modeling of EBV populations has yet to be well defined, especially compared to genomic virus mutations. For this reason, we assessed the contribution of both events (recombination and mutations) in the definition of different EBV populations.

### Impact of recombination and mutation in the EBV genome

The relative impact of recombination on phylogenomic inference accuracy is still a neglected field^59^; especially in EBV where this could explain for lack of consensus on its classification. For a reliable number of sequences to analyze recombination on the EBV phylogenetic inferences, we used a novel approach based on identifying and masking the recombinant regions with Ns (ClonalFrameML analysis). ClonalFrameML identified 432 putative recombination events in the whole genome, of which 85 were found in single genomes (~20%), and 215 events in ancestral nodes (~50%). Results showed a non-uniform distribution of putative recombination events along the genome (Supplementary material, Fig. S1 and Table S5). However, a higher density of putative recombination segments was observed in specific clades, and some isolates had a higher number of putative recombinant regions. Interestingly, genes of certain regions such as those located in the 3′-end and center of the genome, had a greater number of putative recombinant regions.

Regarding the three geographic clades identified in this study, the Kenya I Clade (node 158) had 81% bases in the clonal frame and 4 recombinant regions containing 129 of 351 single nucleotide polymorphism (SNPs); the Kenya II Clade (node 125) had 94% bases in the clonal frame and no recombinant regions; Australia-UK Clade (node 47) had 82% in the clonal frame, with 2 recombinant regions containing 150 of 229 SNPs (Supplementary material, Fig. S2 and Table S6).

To understand which force (mutation or recombination) influences more strongly the EBV genome diversity, values calculated show the relative rate of recombination to mutation (R/θ) was 0.4343, the average length of the recombined fragment (δ) was 661 bp, and average distance between donor and recipient (ν) was 0.0087. Therefore, the relative recombination and mutation effect was estimated in 2.5 (r/m = R/θ × δ × ν), strongly suggesting that recombination events are 2.5-fold more frequent than *de novo* mutations in EBV.

Gubbins analysis identified putative recombination regions in the EBV genome similar to ClonalFrameML, but with clearer definition of ancestry in the recombinant regions (Supplementary material, Fig. S3). Gubbins estimated that, among the 186 ancestral nodes contained in the genome tree, 102 (~55%) evidenced recombination distributed from 1 to 4 putative regions. Among these 102 nodes, 63 showed recombination in only one putative recombinant region, 25 in 2, 11 nodes in 3 and 3 nodes in 4 putative recombinant regions. Similar to ClonalFrameML, both mutations and recombination were found within the whole EBV genome, however, recombination were more frequent.

In addition, Gubbins identified that most recombination events occur in multiple isolates and few recombination patterns are unique from one isolate. Most of the recombinant regions concentrated in a specific genomic location (i.e., the central area); however, surprisingly, they were clustered by phylogenetic clades, suggesting that most of the regions (shared by multiple isolates), were acquired from the node ancestor (Supplementary material, Fig. S3). For instance, we observed 43 visible recombination regions shared by a specific node within the genomic tree, whereas other nodes shared 1 to 3 recombinant regions, one being the most frequent. Five other nodes showed two recombinant regions: node 23 (R2 and R7), node 24 (R3 and R6), node 90 (R15 and R17), node N153 (R30 and R33) and node 158 (R37 and R38), while only one evidenced three recombination regions N140 (R24 R25 and R26) (Supplementary material, Fig. S2 and Table S6). The R5 recombinant region was present in most isolates; thus, it is likely an important acquisition among recombinant regions.

**Figure 3:**
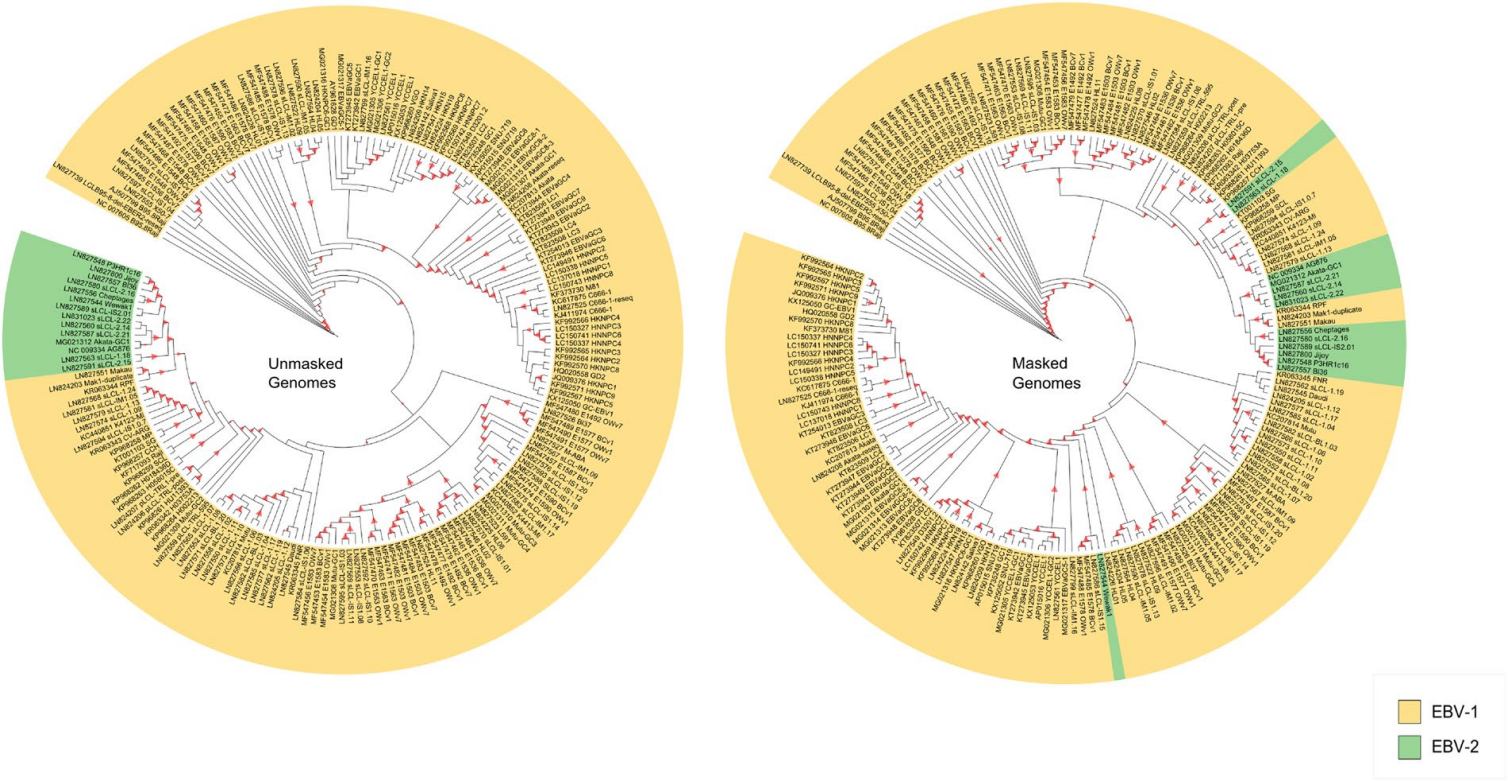
Comparison of phylogenetic trees from unmasked genomes and masked genomes. ML tree based on sequences from the original genome alignment (with putative recombination) and masked alignment (without recombination). The isolates classified as EBV-2 are shown in green and those classified as EBV-1 are represented in yellow. The red triangles indicate bootstrap values above 80%. The traditional classification was recovered only in the unmasked tree; the masking process of recombinant regions in entire genome tree made it possible to re-examine the previously identified EBV-1 and EBV-2 clusters.

The high number of isolates belonging to a clade sharing recombination fragments with a common ancestor indicates that recombination could alter the EBV population structure. In understanding the impact of recombination on the EBV phylogenetic reconstruction, the ML trees of entire genome (with masked recombinant regions) were compared to the unmasked original genome alignment. Results showed a remarkable recovery of EBV type 2 cluster in the original tree and appeared to be dissociated into four different monophyletic clades, meanwhile the Wewak isolate was not contained in a specific clade in the masked tree (Fig. 3). These results suggest that the fact that some strains appear classified as EBV type 2 is a product of recombination events.

**Figure 4:**
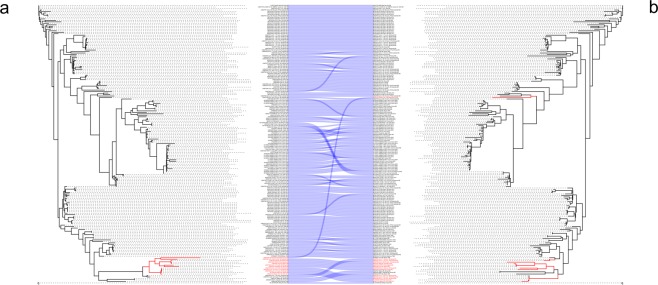
Comparison of isolates positions in entire unmasked and masked genome phylogeny. ML tree (**a**) Unmasked genome (sequences with putative recombination sites) and (**b**) masked genome tree alignment (with recombinant regions masked). The blue lines connect the identical isolates according to their location in each tree. The isolates classified as EBV-2 by the traditional classification are shown in red. Some isolates presented a slight position change between the two trees without affecting their phylogenetic relationship with other isolates. However, other isolates showed radical changes, deeply affecting their association with other isolates.

To understand how recombination affects phylogenetic reconstruction of EBV genomes, isolate position in clades, belonging to an unmasked ML tree (containing recombinant portions, Fig. 3, on the left) were compared to those from a masked tree (recombinant portions masked, Fig. 3, on the right). When comparing both trees, most isolates showed a similar topology (Fig. 4, the dotted blue lines). Some isolates slightly changed, with a different position in the same clade, whereas other isolates had a drastic change, located in different or very distant clades. These results demonstrate the impact of recombination on different levels of EBV evolution.

### A comparison of masked and unmasked EBV phylogenies according to geographical origin

Masked and unmasked trees compared showed that the three clades previously identified here as Australia-UK, Kenya I and Kenya II were also present in the masked tree. However, the main result shows that isolates classified as EBV-2 belong in fact to different clades (Fig. 3). The clades Kenya III (sLCL-2.15 and sLCL-1.18), Kenya IV (Cheptages and sLCL-2.16), Africa-USA (sLCL-2.14, sLCL-2.21, sLCL-2.22, AG876 and Akata-GC1) and Africa-Australia (sLCL-IS2.01, P3HR1 and Jijoye) were present in all trees with high support.

All these results suggest that the traditional classification of EBV reflects a wide range of recombination events acquired over many years, in some genomic regions that segregates EBV into type 1 and type 2.

### Defining the EBV phylogroups

**Figure 5:**
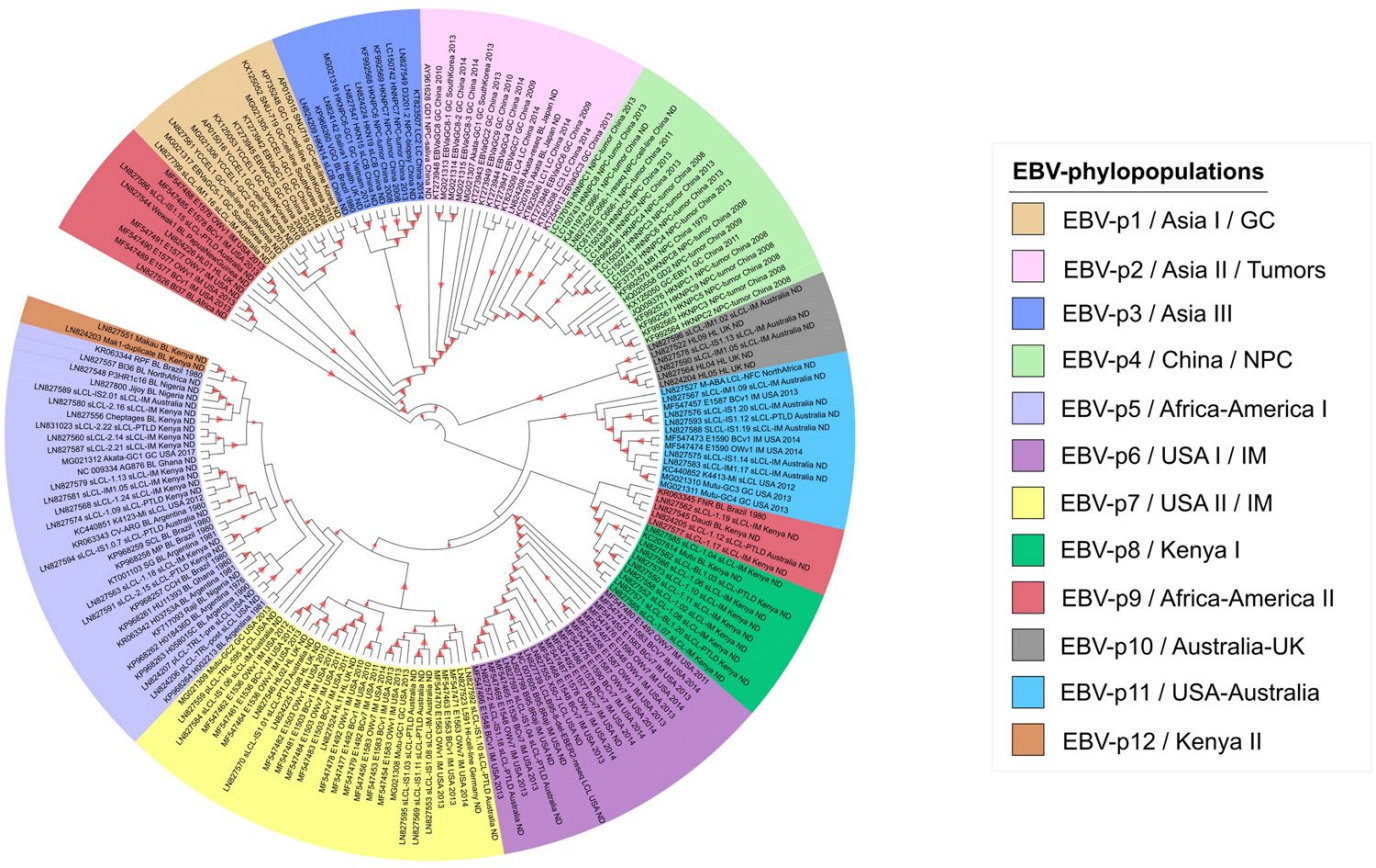
Overlap between population and phylogenetic groups of masked genomes. Branches are colored according to the 12 EBV-phylopopulation identified. The red triangles indicate bootstrap values above 80%. BL - Burkitt’s lymphoma, GC - gastric cancer, HL - Hodgkin’s lymphoma, IM - infectious mononucleosis, LC - lung carcinoma, NPC - nasopharyngeal carcinoma and PTLD - post-transplant lymphoproliferative disease

Previous EBV classification systems, such as type 1 and 2, LMP1 variants and EBNA-1 strains, are significantly simple, considering only specific haplotypes or single genes. However, as these systems do not consider the recombination effect, the EBV phylogenetic structure is unclear. Appropriately, we propose a new classification obtained through alignments of masked genomes using the Bayesian analysis of population structure (hierBAPS). This analysis identified 12 EBV population groups that, along with the phylogenomic tree, constitute this new classification called EBV-phylopopulation (EBV-p) (Fig. 5). Clustering results demonstrate broad agreement between population and phylogenetic cluster. Most population groups displayed monophyletic origins: EBV-p3, EBV-p4, EBV-p6, EBV-p8, EBV-p10, EBV-p11 and EBV-p12, with the exception of EBV-p1,  EBV-p2, EBV-p5, EBV-p7 and EBV-p9 being paraphyletic. No comparable patterns were found when CDS trees were represented according these phylopopulations (Fig. S4).

As depicted in Fig. 3, it is important to note that phylogeny alone cannot distinguish some population groups due to the presence of paraphyletic groups. Thus, a populational detection approach was applied in order to resolve the clades not satisfying the monophyly condition. For example, EBV-p2 could only be discriminated from the EBV-p4 by a populational analysis. Similarly, EBV-p5 does not include Mutu-GC2 (MG021309), Makau BL (LN827551) and Mak1 BL (LN824203), where the former belongs to EBV-p7 and latter to EBV-p12.

### The EBV phylopopulations and their relationship with geographical source and disease

Despite many studies having tried to associate the EBV classification with geographic source and disease progression^15^, these still remain unclear. In this regard, we used those statistically-supported clades from the masked genome tree classified by population group in order to assess any kind of geographical or clinical association. We found four clades EBV-p1 and EBV-p3 with up to 80% of isolates; and EBV-p2 and EBV-p4 with all isolates containing mostly sequences from Asia (Fig. 5). For this reason, these groups could also be called Asia I (EBV-p1), Asia II (EBV-p2), Asia III (EBV-p3), and China (EBV-p4, because they contain sequences exclusively observed from China,1980 to 2013). EBV-p8 and EBV-p12 had sequences solely from Kenya and could be called Kenya I and Kenya II, respectively. EBV-p6 and EBV-p7 had more than 65% of the sequences from the United States and could be called USA I and USA II, respectively. EBV-p5 and EBV-p9 had approximately half of the sequences from Africa and half from America, and could be called Africa-America I and Africa-America II, respectively. EBV-p10 had approximately half of the sequences from Australia and half from the United Kingdom and could be called Australia-UK. EBV-p11 had approximately half of the sequences from the United States and half from Australia and could be called USA-Australia. Even considering the bias in the sampling of EBV genomes, these results suggest that some EBV populations have a markedly geographical distribution.

Additionally, four populations were associated with tumor development: EBV-p1/Asia I with gastric cancer (GC) (except sLCL-IM1.06 isolate associated with infectious mononucleosis, IM), EBV-p2/Asia II isolates associated with tumor diseases including nasopharyngeal cancer (NPC), gastric cancer (GC), Burkitt’s lymphoma (BL), lung carcinoma (LC) and EBV-p4/China associated with NPC (except GC-EBV1 isolate associated with GC). Moreover, two populations, EBV-p6/USA I (up to 80% isolates) and EBV-p7/USA II (up to 60% of isolates) were mostly related to IM.

These results suggest that the phylopopulations identified constitute an interesting way to study, characterize and monitor the different EBV lineages in terms of geographical location or clinical manifestations, leading to better epidemiological surveillance of EBV. However, it is necessary to increase efforts to sequence more complete EBV genomes, especially from different geographical locations and disease conditions.

## Discussion

EBV infects more than 90% of the human population but, causes illness in limited percentage of people^60^. In fact, more than 1% of human cancers worldwide are associated with EBV^61^. In this regard, most of the EBV studies have a clinical approach, evading both the understanding of the pathogen *per se* and the genetic focus of the virus. In the past, methodology limitations made the study of EBV diversity restricted to a unique genome during about 20 years^62^. Fortunately, thanks to advances in technology more EBV genomes from diverse geographical areas and from several types of primary tumor have been sequenced^22,63,64^.

The most used EBV classification establishes two types, EBV-1 and EBV-2, based on alleles of EBNA2 and EBNA3s genes. However, the availability of additional public genomes has promoted the emergence of new classifications by using other EBV genomic regions. The controversy among the different EBV classification systems has been raised because these schemes are not concordant with each other. The questions arising every time when working with EBV genomes are: Can we arbitrarily decide which classification to use? or Can several classifications be used at the same time?

In this sense, some authors have suggested that EBV phylogeny history should not be treated with a reductionist approach. In fact, Choi *et al*.^65^ emphasize the need of reestablishment of phylogenetic EBV relationships using genome-wide approaches to better infer the relationship between EBV and EBV-associated diseases^65^. In order to better understand the genetic history of EBV, we have explored the phylogenetic relationship among 188 isolates using data from whole viral genomes and nine protein-coding CDSs that are pivotal for the viral cycle. These nine CDS are selected because, according to the literature, they are the most involved in latent and lytic cycle^26,52,53^, however some of these genes are colocalized in the genome and probably do not reflect the recombination events happened along the genome.

The recombinant regions within EBV genome were assessed to explore the impact that these recombinant regions have on EBV phylogenies and DNA mutations. The reconstruction of the ancestral history of an organism is usually made by phylogenetic methods that have a great power to infer the evolutionary history; nevertheless, this recombination could distorts the phylogenetic inferences in several points, inducing a non-reliable reconstruction of the tree and, thus, increasing the difficulty of typing EBV strains when a single-gene tree is used to explain the virus evolutionary story^66^.The predicted recombinant regions demonstrated to generate a background interference in the phylogenies, thus, when these regions were masked, the population structure of EBV was revealed.

The sequenced EBV genomes often have artificially-generated nucleotide changes produced either during the genome sequencing, or sequence assembly process^13^, or during the passing process of cell lines in the laboratory. For instance, Palser *et al*.^22^ had to blanked out more than 20 repeat regions from 71 virus genomes to increase the assembly accuracy of repetitive region and thus facilitate comparisons between the strains. Previous studies have shown that cell passages have scarce influence to change the EBV copy number within EBV-transformed lymphoblastoid cell lines^67^; however, this viral culture could induce small artificial nucleotide changes in the EBV genome^13^.

On this regard, our dataset excludes the poorly aligned sites and extreme divergent regions to explore genomes with reliable genomic content, and also assess the available viral replicates and derivative strains. Moreover, those poorly aligned sites were ignored with the purpose to prevent the generation of artificial genomic diversity and avoid assigning a greater phylogenetic weight to certain strains.

A comparison of the different phylogenetic trees obtained yielded incongruent topological results, similar to those found in the literature^22,33,63,68^. To tackle this issue, we compute the genetic distance among the genomes and thus, it was possible to sort EBV according to the traditional classification, showing that Jijoye and the sequences of its derivatives P3HR1^69^, BL36, sLCL-2.16, Cheptages, Wewak1, Akata-GC1, AG876, sLCL-IS2.01, sLCL1.18, sLCL-2.1, sLCL-2.15, sLCL2.21 and sLCL-2.224 belong to EBV type 2, while the remaining 174 isolates belong to EBV type 1.

Previous studies have shown that EBV type 1 and 2 could be assessed by Principal Component Analysis (PCA) based on the sequences of the EBNA2 and EBNA3 genes suggesting a strong genetic linkage of these two genes^22,28^. Interestingly, the EBNA2 gene, which has been widely used to recover the traditional classification of EBV, cold not distinguish all the members initially belonging to the EBV-2 group. Kim *et al*.^70^ showed that some naturally occurring strains of EBV are genotypically discordant by using EBNA2 and EBNA3 genes, as well as Palser *et al*.^22^ who showed that BL36 and sLCL 1.18 are intertypic recombinant virus. All these strains are commonly classified in different types according to EBNA2 and EBNA3 genes, but they are actually intertypic recombinants. These results, along with the inconsistencies in the comparison of topologies obtained from the nine CDS and the whole genome, reinforce the idea that recombination has a strong influence on EBV phylogenies.

A large-scale analysis was performed, searching for recombination in the EBV genome based on the literature data^13,22,63^, with several recombinant regions being identified in EBV strains. More importantly, we found these recombination events occurred 2.5-fold more often than mutations events, suggesting that the impact of recombination on virus genomic diversity is much more important than mutation events. Moreover, most of these recombination regions were detected within ancestral node positions in a particular array, indicating that some current lineages have been shaped by recombination. There were some regions in the genome with acquisition hotspot of alternative sequences (Repeat regions^11^ and some genes like EBNA3A^54^) as a possible result of the immune system pressure to adaptation. These recombination events depend on the coinfection by different types of EBV within the same cell^30,54^. Our results suggest that recombination is an evolutionary force that could trigger not only genomic modifications, but also an increase of virus pathogenicity or the spreading these traits to new variants (with new haplotypes) which cold impact the EBV population structure.

Considering the impact of recombination on the evolutionary history of EBV, we decided to take an innovative approach to studying EBV evolution, removing the identified recombinant regions from genomes (replacing these regions with Ns) and comparing phylogenetic reconstructions of genomes with and without recombination sites. In this regard, we demonstrated that recombination resulted in changes from discrete (within the same clade) to radical (among distant clades) along the phylogenetic relations of some studied isolates.

In spite of the strong evidence that recombination has played a crucial role in the evolution of the EBV genome, as stated above, these findings are not enough to define which would be the most accurate criterion to classify EBV diversity. Thus, we analyzed the population structure of the virus using the Bayesian analysis of population structure (hierBAPS) approach to define the reability of those populations not phylogeneticly solved.

The population result managed to overlap the topology of the genome tree without the recombination regions, defining the 12 populations, of those seven were monophyletic and five paraphyletic. The paraphyly phenomenon was possibly due to the lack of polymorphisms or phylogenetic signal to be defined as monophyletic clades. EBV diversity is evidenced by its tree topology, which usually has a considerable number of paraphyletic clades making it even more difficult to define clades and establish a proper classification. Therefore, EBV diversity is the result of an extensive succession of recombination events that have been occurring over a prolonged period because many recombination regions were obtained from their ancestors (see Supplementary material, Fig. S3).

As suggested in previous reports^71,72^, EBV has a geographical structure different from others human herpesviruses, such as HHV-6A and HHV-6B^73^. Based on the populations defined here, we were identified populations associated to both geographical location and certain diseases. In this sense, four population are directly related to Asia, with distinct spatial-temporal distribution (samples from China, Japan, Korea and Vietnam collected between 1970 and 2014), indicating a marked circulation of diverse EBV populations among Asian countries. In addition, two populations have been exclusively circulating in Kenya, this conclusion could be bias, because of the sample number. Whereas the other six EBV populations have shown a broader geographical circulation, possibly because these populations are the most strongly associated with the high prevalence of EBV in the world.

Interestingly, among the Asiatic populations: a EBV-p1/Asia I population was more frequently associated with gastric cancer, being the only exception the Australian isolate sLCL-1.16 that was from a patient with infectious mononucleosis (IM). Similarly, the EBVp4/China was more frequently associated with nasopharyngeal carcinoma (NPC), being the only exception the GC-EBV1 isolate, which belongs to a gastric cancer patient that might have developed the EBV non-associated gastric cancer. On the other hand, the EBV-p2/Asia II population also showed association with several types of tumors and lymphoproliferative disorders (LPD).

Chiara *et al*.^71^ described seven sub-populations of EBV using 127 genomes, showing an association with geographic location. However, our study based on 188 genomes contrasts with their findings because in our analyses the recombinant regions were removed, revealing a more complex population structure consisting of 12 identified populations. In this way, some isolates that were classified as a unique population by Chiara *et al*.^71^ were classified as belonging to different populations according to our analysis. For instance, the population G5 according to Chiara *et al*.^71^ corresponds to the population EBV-p7/USA II/IM and EBV-P11/USA-Australia in our classification. Furthermore, some populations in our study were correlated with some diseases (GC, NPC and IM).

However, our study was limited by the bias in the available EBV genome sequences resulting in a small number of EBV type 2 isolates and lack of EBV isolates in certain phylopopulations. To solve this issue is necessary to add new sequence genomes from different geographic locations and from individuals with different clinical manifestation. Even though this limitation, here we propose a reliable approach for EBV classification in order to clarify the population structure, resolve the paraphyly phenomenon found in phylogenies and suggest the relationship between these populations and EBV-related diseases.

In the present study, we explored the EBV historical evolution by combining population and phylogenomic tools to define the population structure in order to contribute in the understanding of the relationship among the EBV population, its geographical origin, the implication of certain strains in diseases and the potential application of this knowledge to develop diagnostic tools and epidemiological surveillance of this virus.

Our findings indicate that recombination plays a crucial role in the diversification of EBV, clearly more than mutations. A considerable number of recombinant regions were acquired by the current strains from their ancestors. Therefore, the classification of EBV is not simplistic and the most consistent manner to classify this virus should be based on EBV populations with masked recombinant regions, which reliably reflect the evolutionary history of the virus.

## Supplementary information

Supplementary Information

Supplementary dataset
