# Supplementary material for: A reliable Epstein-Barr Virus classification based on phylogenomic and population analyses
Source: Sci Rep. 2019 Jul 8;9:9829. doi: 10.1038/s41598-019-45986-3 (PMC6614506; doi:10.1038/s41598-019-45986-3)
Supplement: Supplementary file 1 — Supplementary Information [file 41598_2019_45986_MOESM1_ESM.docx]

**Supplementary information**

**A reliable Epstein-Barr Virus classification based on phylogenomic and population analyses**

Louise Zanella^1,2,3†^, Ismael Riquelme^4†^, Kurt Buchegger^1,2,3^, Michel Abanto^3^, Carmen Ili^1,2,3*^, Priscilla Brebi^1,2,3*^

^1^. Laboratory of Molecular Pathology, Department of Pathology, School of Medicine, Universidad de La Frontera, Temuco, Chile.

^2^. Center for Excellence in Translational Medicine (CEMT), Universidad de La Frontera, Temuco, Chile.

^3^. Scientific and Technological Bioresource Nucleus (BIOREN), Universidad de La Frontera, Temuco, Chile.

^4^ Instituto de Ciencias Biomédicas, Facultad de Ciencias de la Salud, Universidad Autónoma de Chile, Chile.

^†^ These authors contributed equally to this work.

* To whom correspondence should be addressed.

Dr. Priscilla Brebi Phone: +56 45 2596583; E-mail: priscilla.brebi@ufrontera.cl

Dr. Carmen Ili: +56 45 2596693; E-mail: carmen.ili@ufrontera.cl

Email addresses:

LZ: louise.zanella@ufrontera.cl

IR: ismael.riquelme.contreras@gmail.com

KB: k.buchegger@gmail.com

MA: michel.abanto@ufrontera.cl

**Supplementary Table S1** List of 188 genome sequences used in this study

| Isolate | Accession number |
| --- | --- |
| B95.8Raji | NC_007605 |
| AG876 | NC_009334 |
| YCCEL1 | AP015016 |
| M81 | KF373730 |
| Cheptages | LN827556 |
| sLCL-IS1.20 | LN827576 |
| sLCL-2.16 | LN827580 |
| sLCL-IM1.02 | LN827596 |
| sLCL-2.14 | LN827560 |
| sLCL-2.21 | LN827587 |
| sLCL-IS1.13 | LN827578 |
| Jijoy | LN827800 |
| sLCL-IS1.14 | LN827575 |
| sLCL-2.15 | LN827591 |
| sLCL-IM1.05 | LN827590 |
| sLCL-2.22 | LN831023 |
| Daudi | LN827545 |
| P3HR1c16 | LN827548 |
| sLCL-IM1.09 | LN827567 |
| sLCL-IM1.17 | LN827583 |
| sLCL-IS2.01 | LN827589 |
| HKN14 | LN824209 |
| SLCL-IS1.19 | LN827588 |
| HL11 | LN827524 |
| Wewak1 | LN827544 |
| Bl36 | LN827557 |
| HKN15 | LN827547 |
| HKN19 | LN824224 |
| sLCL-IS1.01 | LN827570 |
| sLCL-IS1.11 | LN827569 |
| sLCL-IS1.10 | LN827592 |
| sLCL-IS1.18 | LN827572 |
| sLCL-IS1.12 | LN827593 |
| EBVaGC1 | KT273942 |
| M-ABA | LN827527 |
| HL04 | LN827564 |
| sLCL-1.12 | LN824205 |
| sLCL-IS1.0.7 | LN827594 |
| HL09 | LN827522 |
| D3201.2 | LN827549 |
| YCCEL1 | LN827561 |
| sLCL-BL1.03 | LN827582 |
| Bl37 | LN827526 |
| sLCL-1.19 | LN827562 |
| sLCL-IS1.15 | LN827586 |
| C666-1-reseq | LN827525 |
| sLCL-IM1.05 | LN827581 |
| sLCL-1.11 | LN827550 |
| sLCL-1.18 | LN827563 |
| sLCL-1.06 | LN827566 |
| sLCL-BL1.20 | LN827571 |
| Isolate | **Accession number** |
| sLCL-1.04 | LN827585 |
| sLCL-IM1.16 | LN827799 |
| sLCL-1.17 | LN827577 |
| sLCL-1.08 | LN827552 |
| sLCL-1.24 | LN827568 |
| HL02 | LN827546 |
| HL08 | LN824225 |
| sLCL-1.10 | LN827573 |
| LCLB95-8-del-EBER2-reseq | LN827739 |
| sLCL-1.07 | LN827565 |
| RPF | KR063344 |
| sLCL-1.02 | LN827558 |
| sLCL-IS1.08 | LN827553 |
| EBVaGC9 | KT273949 |
| pLCL-TRL-595 | LN827559 |
| K4413-Mi | KC440852 |
| Mak1-duplicate | LN824203 |
| sLCL-IS1.06 | LN827584 |
| FNR | KR063345 |
| H03753A | KR0633420 |
| H002213 | KP968264 |
| B95.8Raji | AJ507799 |
| H058015C | KP968263 |
| CV-ARG | KR063343 |
| SCL | KP968259 |
| X50-7 | LN827555 |
| SG | KT001103 |
| HU11393 | KP968261 |
| HL01 | LN824226 |
| H018436D | KP968262 |
| K4123-Mi | KC440851 |
| sLCL-1.13 | LN827579 |
| sLCL-IS1.03 | LN827595 |
| HL05 | LN824204 |
| HNNPC5 | LC150338 |
| sLCL-1.09 | LN827574 |
| CCH | KP968257 |
| sLCL-IS1.04 | LN827597 |
| EBVaGC2 | KT273943 |
| EBVaGC5 | KT273945 |
| Akata-reseq | LN824208 |
| LC3 | KT823508 |
| Saliva1 | LN824142 |
| Makau | LN827551 |
| pLCL-TRL-post | LN824206 |
| MP | KP968258 |
| Mutu | KC207814 |
| EBVaGC7 | KT273947 |
| VGO | KP968260 |
| EBVaGC8 | KT273948 |
| Isolate | **Accession number** |
| EBVaGC4 | KT273944 |
| GD1 | AY961628 |
| LC2 | KT823507 |
| HNNPC2 | LC149491 |
| EBVaGC3 | KT254013 |
| EBVaGC6 | KT273946 |
| LC4 | KT823509 |
| L591 | LN827523 |
| pLCL-TRL1-pre | LN824207 |
| LC1 | KT823506 |
| HKNPC3 | KF992565 |
| HKNPC6 | KF992568 |
| HKNPC1 | JQ009376 |
| HNNPC6 | LC150741 |
| HNNPC4 | LC150337 |
| HKNPC8 | KF992570 |
| HNNPC1 | LC137018 |
| HKNPC5 | KF992567 |
| Akata | KC2078130 |
| C666-1 | KC617875 |
| HKNPC4 | KF992566 |
| HNNPC7 | LC150742 |
| HNNPC3 | LC150327 |
| C666-1 | KJ411974 |
| HKNPC9 | KF992571 |
| GC1 | KP735248 |
| HKNPC7 | KF992569 |
| SNU-719 | KX125052 |
| HKNPC2 | KF992564 |
| HNNPC8 | LC150743 |
| GC-EBV1 | KX125050 |
| SNU719 | AP015015 |
| YCCEL1 | KX125053 |
| Raji | KF717093 |
| GD2 | HQ020558 |
| E1583_BCv1 | MF547453 |
| E1583_OWv1 | MF547454 |
| E1583_BCv7 | MF547455 |
| E1536_OWv7 | MF547464 |
| E1536_BCv7 | MF547465 |
| E1548_BCv1 | MF547466 |
| E1548_OWv1 | MF547467 |
| E1548_BCv7 | MF547468 |
| E1548_OWv7 | MF547469 |
| E1563_OWv1 | MF547470 |
| E1563_OWv7 | MF547471 |
| E1563_BCV7 | MF547472 |
| E1590_BCv1 | MF547473 |
| E1590_OWv1 | MF547474 |
| E1590_BCv7 | MF547475 |
| E1590_OWv7 | MF547476 |

| Isolate | Accession number |
| --- | --- |
| E1492_BCv1 | MF547477 |
| E1492_OWv1 | MF547478 |
| E1492_BCv7 | MF547479 |
| E1492_OWv7 | MF547480 |
| E1503_BCv1 | MF547481 |
| E1503_OWv1 | MF547482 |
| E1503_BCv7 | MF547483 |
| E1503_OWv7 | MF547484 |
| E1578_BCv1 | MF547485 |
| E1578_BCv7 | MF547486 |
| E1578_OWv7 | MF547487 |
| E1578_OWv1 | MF547488 |
| E1577_BCv1 | MF547489 |
| E1577_OWv1 | MF547490 |
| E1577_OWv7 | MF547491 |
| E1577_BCv7 | MF547492 |
| EBVaGC8-2 | MG021314 |
| YCCEL1-GC1 | MG021305 |
| EBVaGC8-3 | MG021315 |
| EBVaGC5-1 | MG021317 |
| Mutu-GC1 | MG021308 |
| Akata-GC1 | MG021307 |
| AG876-GC1 | MG021312 |
| HKNPC6-GC1 | MG021316 |
| Mutu-GC3 | MG021310 |
| Mutu-GC4 | MG021311 |
| Mutu-GC2 | MG021309 |

**Supplementary Table S2** Nucleotide substitution models selected

| Region | Substitution  model | Site  heterogeneity |
| --- | --- | --- |
| Genome | GTR | G + I |
| BLLF1 (coding isoform of gp350) | HKY | G + I |
| BZLF1 (exon 1-3) | GTR | G + I |
| EBNA1 | HKY | G + I |
| EBNA2 | GTR | G |
| EBNA3A (exon 1-2) | GTR | G + I |
| EBNA3B (exon 1-2) | GTR | G + I |
| EBNA3C (exon 1-2) | GTR | G + I |
| LMP1 (exon 1-3) | GTR | G + I |
| LMP2 (coding isoform A, exon 1-8) | GTR | G + I |

Based on Akaike information criterion

**Supplementary Table S3** List of isolates of each putative recombinant event identified by RDP4.

| Event | Sequence names |
| --- | --- |
| 1 | Raji |
| 2 | AG876, Cheptages, sLCL-2.16, sLCL-2.14, sLCL-2.21, Jijoy, sLCL-2.22, P3HR1c16, Wewak1, Bl36, sLCL-1.18, RPF, SCL, Akata-GC1 |
| 3 | L591, HL11, sLCL-IS1.11, sLCL-IS1.10, sLCL-IS1.08, sLCL-IS1.03, E1583_BCv1, E1583_OWv7, MF547477, E1492_OWv1, E1492_BCv7, E1503_BCv1, E1503_OWv1, E1503_BCv7, E1503_OWv7 |
| 4 | sLCL-IS2.01, M81, HKN15, HKN19, sLCL-IS1.15, C666-1-reseq, EBVaGC9, Mak1-duplicate, HNNPC5, EBVaGC2, Akata-reseq, LC3, Makau, EBVaGC7, EBVaGC4, GD1, HNNPC2, EBVaGC3, EBVaGC6, LC4, LC1, HKNPC3, HKNPC1, HNNPC6, HNNPC4, HKNPC8, LC137018, HKNPC5, Akata, C666-1, HKNPC4, HNNPC3, C666-1, HKNPC9, HKNPC2, HNNPC8, GD2, Akata-GC1 |
| 5 | GC1, EBVaGC8, SNU-719, SNU719, EBVaGC8-2, EBVaGC8-3, EBVaGC5-1, EBVaGC8-1 |
| 6 | HL08, sLCL-IS1.01, HL02, E1536_OWv1 |
| 7 | HKNPC6, HKNPC7 |
| 8 | sLCL-1.19 |
| 9 | 3002213 |
| 10 | D3201.2, HKN15, HKN19, LC2, HKNPC6, HNNPC7, HKNPC7 |
| 11 | sLCL-2.16, Cheptages, Jijoy, P3HR1c16, Bl36 |
| 12 | E1578_OWv1, EBVaGC4 |
| 13 | EBVaGC4, M81, C666-1-reseq, EBVaGC9, HNNPC5, EBVaGC2, Akata-reseq, LC3, EBVaGC7, HNNPC2, EBVaGC3, EBVaGC6, LC1, HKNPC3, HKNPC1, HNNPC6, HNNPC4, HKNPC8, LC137018, HKNPC5, Akata, C666-1, HKNPC4, HNNPC3, C666-1, HKNPC9, HKNPC2, HNNPC8, GC-EBV1, GD2, Akata-GC1 |
| 14 | EBVaGC9, AP015016, sLCL-IM1.02, sLCL-IS2.01, HL04, HL09, YCCEL1, HL05, HNNPC5, EBVaGC2, EBVaGC5, Akata-reseq, EBVaGC7, EBVaGC8, EBVaGC4, GD1, LC1, Akata, GC1, SNU-719, SNU719, YCCEL1, EBVaGC8-2, YCCEL1-GC1, EBVaGC8-3, EBVaGC5-1, Akata-GC1, HKNPC6-GC1, EBVaGC8-1, YCCEL1-GC2 |
| 15 | EBVaGC3, EBVaGC9, EBVaGC2, EBVaGC5, Akata-reseq, LC3, EBVaGC7, EBVaGC8, EBVaGC4, LC4, LC1, Akata, GC1, SNU-719, SNU719, E1578_BCv1, E1578_OWv1, EBVaGC8-2, EBVaGC8-3, EBVaGC5-1, Akata-GC1, EBVaGC8-1 |
| 16 | HKNPC2, HKN15, HKN19, D3201.2, EBVaGC9, EBVaGC2, Akata-reseq, LC3, EBVaGC7, EBVaGC4, LC2, EBVaGC3, LC4, LC1, HKNPC6, Akata, HNNPC7, HKNPC7, Akata-GC1 |
| 17 | RPF |
| 18 | sLCL-IS1.0.7 |
| 19 | HKNPC6-GC1 |
| 20 | sLCL-IM1.16, M81, sLCL-IS1.13, HKN14, HKN15, HKN19, EBVaGC1, D3201.2, sLCL-1.19, sLCL-IS1.15, C666-1-reseq, LC3, Saliva1, VGO, LC2, HNNPC2, EBVaGC3, EBVaGC6, LC4, L591, HKNPC3, HKNPC6, HKNPC1, HNNPC6, HNNPC4, HKNPC8, HKNPC5, C666-1, HKNPC4, HNNPC7, HNNPC3, C666-1, HKNPC9, HKNPC7, HKNPC2, GC-EBV1, GD2, E1578_BCv1, E1578_OWv1 |
| 21 | E1548_OWv1, B95.8Raji, AG876, sLCL-2.14, sLCL-2.21, sLCL-2.22, sLCL-IM1.05, sLCL-1.24_, LCLB95-8-del-EBER2-reseq, Mak1-duplicate, H03753A, H002213, B95.8Raji, X50-7, HU11393, K4123-Mi, sLCL-1.13, sLCL-1.09, CCH, sLCL-IS1.04, Makau, E1583_BCv7, E1587_OWv1, E1587_OWv7, E1536_BCv1, E1536_OWv1, E1536_OWv7, E1536_BCv7, E1548_BCv7, E1548_OWv7, E1563_BCV7, E1590_BCv7, E1590_OWv7, E1492_OWv7, E1578_BCv7, E1578_OWv7, E1577_BCv7, Akata-GC1 |
| 22 | pLCL-TRL-post, H002213, pLCL-TRL1-pre, Mutu-GC2 |
| 23 | sLCL-IM1.02, sLCL-IS1.01, sLCL-IS1.10, HL09, HL02, HL08, sLCL-IS1.08, sLCL-IS1.03, E1583_BCv1, E1583_OWv7, E1492_OWv1, E1503_OWv1 |
| 24 | VGO, Saliva1 |
| 25 | Akata-GC1, EBVaGC9, EBVaGC2, Akata-reseq, EBVaGC7, EBVaGC4, GD1, LC1, Akata |

**Supplementary Table S4** RDP4 methods with supporting p-values for putative recombinant events in genome, EBNA3s genes.

| Event (n) | RDP | Bootscan | GENCONV | MaxChi | Chimaera | SciScan | 3Seq |
| --- | --- | --- | --- | --- | --- | --- | --- |
|  |  |  |  |  |  |  |  |
| Genome | | | | | | | |
| 1 | 7.27 x 10^-115^ | 3.84 x 10^-65^ | 1.71 x 10^-117^ | 6.91 x 10^-51^ | 7.26 x 10^-40^ | 2.32 x 10^-41^ | 6.50 x 10^-11^ |
| 2 | 2.93 x 10^-113^ | 4.13 x 10^-63^ | 1.05 x 10^-110^ | 1.30 x 10^-63^ | 3.26 x 10^-36^ | 4.26 x 10^-59^ | 6.50 x 10^-11^ |
| 3 | 4.91 x 10^-104^ | 3.60 x 10^-69^ | 1.04 x 10^-102^ | 1.10 x 10^-24^ | 1.68 x 10^-14^ | 5.47 x 10^-28^ | 2.10 x 10^-117^ |
| 4 | 1.64 x 10^-87^ | 2.76 x 10^-46^ | 5.54 x 10^-93^ | 4.56 x 10^-42^ | 2.72 x 10^-25^ | 1.94 x 10^-42^ | 1.30 x 10^-10^ |
| 5 | 5.41 x 10^-49^ | 9.39 x 10^-17^ | 8.03 x 10^-48^ | 2.05 x 10^-24^ | 7.36 x 10^-26^ | 3.98 x 10^-23^ | 2.03 x 10^-11^ |
| 6 | 1.56 x 10^-39^ | 4.58 x 10^-05^ | 1.28 x 10^-44^ | 2.51 x 10^-20^ | 1.21 x 10^-19^ | 7.39 x 10^-24^ | 6.45 x 10^-25^ |
| 7 | 4.74 x 10^-43^ | 4.10 x 10^-19^ | 1.01 x 10^-40^ | 8.57 x 10^-14^ | 3.61 x 10^-14^ | 1.32 x 10^-11^ | 6.50 x 10^-11^ |
| 8 | 8.68 x 10^-34^ | 2.94 x 10^-02^ | 5.37 x 10^-39^ | 2.72 x 10^-16^ | 3.03 x 10^-08^ | 1.84 x 10^-22^ | 6.50 x 10^-11^ |
| 9 | 2.59 x 10^-41^ | 8.11 x 10^-11^ | 4.64 x 10^-37^ | 4.00 x 10^-15^ | 3.92 x 10^-14^ | 3.10 x 10^-16^ | 6.50 x 10^-11^ |
| 10 | 4.36 x 10^-32^ | 5.25 x 10^-12^ | 6.55 x 10^-36^ | 3.61 x 10^-14^ | 2.43 x 10^-09^ | 6.75 x 10^-21^ | 6.50 x 10^-11^ |
| 11 | 2.01 x 10^-33^ | 2.15 x 10^-14^ | 4.21 x 10^-34^ | 2.64 x 10^-18^ | 1.96 x 10^-18^ | 5.07 x 10^-21^ | 6.50 x 10^-11^ |
| 12 | 1.84 x 10^-34^ | 5.84 x 10^-13^ | 1.52 x 10^-32^ | 1.87 x 10^-13^ | 5.81 x 10^-14^ | 4.36 x 10^-14^ | 6.50 x 10^-11^ |
| 13 | 3.23 x 10^-38^ | 2.84 x 10^-22^ | 4.08 x 10^-34^ | 4.97 x 10^-12^ | 3.92 x 10^-12^ | 4.68 x 10^-14^ | 6.50 x 10^-11^ |
| 14 | 3.15 x 10^-29^ | 1.27 x 10^-19^ | 1.53 x 10^-27^ | 2.63 x 10^-08^ | 5.85 x 10^-08^ | 4.17 x 10^-09^ | 6.50 x 10^-11^ |
| 15 | 1.01 x 10^-24^ | 4.02 x 10^-12^ | 5.99 x 10^-27^ | 2.41 x 10^-11^ | 7.99 x 10^-09^ | 8.58 x 10^-14^ | 6.50 x 10^-11^ |
| 16 | 1.19 x 10^-28^ | 2.30 x 10^-10^ | 8.45 x 10^-26^ | 1.13 x 10^-07^ | 2.66 x 10^-07^ | 9.62 x 10^-07^ | 6.50 x 10^-11^ |
| 17 | 4.59 x 10^-20^ | 1.73 x 10^-21^ | 1.35 x 10^-23^ | 7.12 x 10^-12^ | 2.53 x 10^-04^ | 4.23 x 10^-04^ | 6.50 x 10^-11^ |
| 18 | 9.72 x 10^-21^ | 1.46 x 10^-13^ | 1.02 x 10^-18^ | 3.26 x 10^-08^ | 1.01 x 10^-07^ | 3.39 x 10^-05^ | 6.50 x 10^-11^ |
| 19 | 2.79 x 10^-20^ | 2.35 x 10^-19^ | 1.79 x 10^-18^ | 4.42 x 10^-08^ | 8.07 x 10^-06^ | 1.10 x 10^-11^ | 4.33 x 10^-03^ |
| 20 | 1.84 x 10^-12^ | 4.99 x 10^-19^ | 6.67 x 10^-19^ | 6.98 x 10^-10^ | 2.80 x 10^-06^ | 1.25 x 10^-16^ | 1.30 x 10^-10^ |
| 21 | 2.61 x 10^-184^ | 1.15 x 10^-18^ | 9.23 x 10^-13^ | 9.86 x 10^-12^ | 3.68 x 10^-12^ | 1.31 x 10^-12^ | 1.97 x 10^-27^ |
| 22 | 4.32 x 10^-12^ | 4.42 x 10^-05^ | 1.23 x 10^-04^ | 4.61 x 10^-10^ | 4.51 x 10^-06^ | 8.08 x 10^-09^ | 6.50 x 10^-11^ |
| 23 | 2.27 x 10^-14^ | 2.63 x 10^-06^ | 1.50 x 10^-13^ | 4.90 x 10^-05^ | 4.50 x 10^-05^ | 4.47 x 10^-03^ | 7.29 x 10^-15^ |
| 24 | 2.34 x 10^-12^ | 5.49 x 10^-12^ | 3.85 x 10^-12^ | 1.62 x 10^-10^ | 8.973 x 10^-11^ | 1.37 x 10^-14^ | 8.90 x 10^-15^ |
| 25 | 9.85 x 10^-15^ | 5.90 x 10^-09^ | 9.76 x 10^-12^ | 6.74 x 10^-10^ | 3.12 x 10^07^ | 7.64 x 10^-07^ | 2.53 x 10^-09^ |
| EBNA3A | | | | | | | |
| 1 | 3.50 x 10^-8^ | 8.65 x 10^-8^ | 1.17 x 10^-4^ | 1.74 x 10^-2^ | 5.93 x 10^-3^ | 3.72 x 10^-34^ | 3.80 x 10^-5^ |
| EBNA3B | | | | | | | |
| 1 | 1.97 x 10^-5^ | 9.37 x 10^-5^ | 1.03 x 10^-3^ | 4.03 x 10^-4^ | 1.51 x 10^-4^ | 3.09 x 10^-8^ | 3.81 x 10^-7^ |

| EBNA3C | | | | | | | |
| --- | --- | --- | --- | --- | --- | --- | --- |
| 1 | 1.12 x 10^-10^ | 3.52 x 10^-3^ | 1.09 x 10^-9^ | 1.68 x 10^-7^ | 8.38 x 10^-8^ | 5.06 x 10^-8^ | 4.85 x 10^-15^ |
| 2 | 3.75 x 10^-6^ | 4.98 x 10^-4^ | 4.61 x 10^-5^ | 6.64 x 10^-7^ | 4.30 x 10^-7^ | 7.05 x 10^-8^ | 6.41 x 10^-10^ |

All values expressed for methods are the p-values with Bonferroni correction

**Supplementary Table S5.** List of reconstructed recombination events identified by ClonalFrameML. Each line indicates one event, the first column indicates the branch (tip or node) on which the event was found. The first and last genomic positions affected by the recombination are also described.

| Tip/Node | ClonalFrameML coordinates | | Coordinates based on NC_007605 | |
| --- | --- | --- | --- | --- |
|  | Beg | End | Beg | End |
| LN827556_Cheptages_BL_Kenya_ND | 6089 | 6777 | 6304 | 7001 |
| LN827556_Cheptages_BL_Kenya_ND | 9981 | 11020 | 63176 | 64225 |
| LN827556_Cheptages_BL_Kenya_ND | 42816 | 44194 | 116637 | 119748 |
| LN827556_Cheptages_BL_Kenya_ND | 47033 | 48742 | 122731 | 124463 |
| LN827556_Cheptages_BL_Kenya_ND | 84868 | 85509 | 195475 | 196189 |
| LN827576_sLCL-IS1.20_sLCL-IM_Australia_ND | 81506 | 85509 | 188608 | 196189 |
| LN827580_sLCL-2.16_sLCL-IM_Kenya_ND | 3866 | 5565 | 4011 | 5749 |
| LN827580_sLCL-2.16_sLCL-IM_Kenya_ND | 49312 | 52121 | 125033 | 127862 |
| LN827580_sLCL-2.16_sLCL-IM_Kenya_ND | 60708 | 62270 | 137704 | 153223 |
| LN827580_sLCL-2.16_sLCL-IM_Kenya_ND | 83750 | 85509 | 193637 | 196189 |
| LN827578_sLCL-IS1.13_sLCL-IM_Australia_ND | 72670 | 72932 | 179486 | 179783 |
| LN827578_sLCL-IS1.13_sLCL-IM_Australia_ND | 82040 | 85509 | 189153 | 196189 |
| LN827591_sLCL-2.15_sLCL-PTLD_Kenya_ND | 10062 | 13930 | 63257 | 67194 |
| LN827545_Daudi_BL_Kenya_ND | 1 | 1879 | 35 | 1917 |
| LN827545_Daudi_BL_Kenya_ND | 14889 | 17692 | 81797 | 84696 |
| LN827545_Daudi_BL_Kenya_ND | 24228 | 27125 | 91389 | 94332 |
| LN827545_Daudi_BL_Kenya_ND | 47363 | 48628 | 123061 | 124349 |
| LN827545_Daudi_BL_Kenya_ND | 63776 | 65627 | 154739 | 156595 |
| LN827567_sLCL-IM1.09_sLCL-IM_Australia_ND | 24959 | 26622 | 92121 | 93807 |
| LN827567_sLCL-IM1.09_sLCL-IM_Australia_ND | 55848 | 57091 | 131682 | 132934 |
| LN827567_sLCL-IM1.09_sLCL-IM_Australia_ND | 68411 | 69244 | 159435 | 160271 |
| LN827567_sLCL-IM1.09_sLCL-IM_Australia_ND | 84278 | 85509 | 194291 | 196189 |
| LN827589_sLCL-IS2.01_sLCL-IM_Australia_ND | 14497 | 15121 | 81395 | 82076 |
| LN827589_sLCL-IS2.01_sLCL-IM_Australia_ND | 43695 | 44163 | 119249 | 119717 |
| LN827589_sLCL-IS2.01_sLCL-IM_Australia_ND | 46988 | 49426 | 122686 | 125147 |
| LN827589_sLCL-IS2.01_sLCL-IM_Australia_ND | 82564 | 85509 | 189765 | 196189 |
| LN824209_HKN14_sLCB_China_ND | 14080 | 16360 | 80733 | 83356 |
| LN824209_HKN14_sLCB_China_ND | 33381 | 35185 | 101239 | 103177 |
| LN824209_HKN14_sLCB_China_ND | 44875 | 46524 | 120496 | 122188 |
| LN824209_HKN14_sLCB_China_ND | 51205 | 52223 | 126931 | 127964 |
| LN827544_Wewak1_BL_PapuaNewGuinea_ND | 1 | 13388 | 35 | 66652 |
| LN827544_Wewak1_BL_PapuaNewGuinea_ND | 18325 | 23976 | 85329 | 91137 |
| LN827544_Wewak1_BL_PapuaNewGuinea_ND | 28560 | 37949 | 95813 | 111162 |
| LN827544_Wewak1_BL_PapuaNewGuinea_ND | 42658 | 43106 | 116479 | 116928 |
| LN827544_Wewak1_BL_PapuaNewGuinea_ND | 50732 | 52947 | 126454 | 128702 |
| LN827544_Wewak1_BL_PapuaNewGuinea_ND | 64922 | 68084 | 155890 | 159108 |
| LN827544_Wewak1_BL_PapuaNewGuinea_ND | 76112 | 79846 | 183042 | 186865 |
| LN827544_Wewak1_BL_PapuaNewGuinea_ND | 82549 | 85509 | 189750 | 196189 |
| LN827557_Bl36_BL_NorthAfrica_ND | 6016 | 7876 | 6231 | 61041 |
| LN827572_sLCL-IS1.18_sLCL-PTLD_Australia_ND | 29737 | 29935 | 97007 | 97715 |
| LN827593_sLCL-IS1.12_sLCL-PTLD_Australia_ND | 84502 | 85509 | 195106 | 196189 |
| KT273942_EBVaGC1_GC_China_2013 | 1 | 472 | 35 | 506 |
| KT273942_EBVaGC1_GC_China_2013 | 15681 | 16499 | 82648 | 83495 |
| KT273942_EBVaGC1_GC_China_2013 | 37313 | 37892 | 106499 | 111105 |
| KT273942_EBVaGC1_GC_China_2013 | 42430 | 42780 | 116251 | 116601 |
| LN824205_sLCL-1.12_sLCL-PTLD_Australia_ND | 1035 | 1544 | 1072 | 1581 |
| LN824205_sLCL-1.12_sLCL-PTLD_Australia_ND | 28970 | 37542 | 96224 | 106728 |
| LN824205_sLCL-1.12_sLCL-PTLD_Australia_ND | 46455 | 48986 | 122119 | 124707 |
| LN824205_sLCL-1.12_sLCL-PTLD_Australia_ND | 82531 | 85509 | 189732 | 196189 |
| LN827582_sLCL-BL1.03_sLCL-PTLD_Kenya_ND | 21391 | 25603 | 88462 | 92781 |
| LN827582_sLCL-BL1.03_sLCL-PTLD_Kenya_ND | 72670 | 73067 | 179486 | 179918 |
| LN827582_sLCL-BL1.03_sLCL-PTLD_Kenya_ND | 77506 | 85509 | 184482 | 196189 |
| LN827526_Bl37_BL_Africa_ND | 12714 | 13371 | 65953 | 66635 |
| LN827526_Bl37_BL_Africa_ND | 50161 | 52223 | 125883 | 127964 |
| LN827526_Bl37_BL_Africa_ND | 84309 | 85509 | 194376 | 196189 |
| LN827562_sLCL-1.19_sLCL-IM_Kenya_ND | 1 | 1069 | 35 | 1106 |
| LN827562_sLCL-1.19_sLCL-IM_Kenya_ND | 16182 | 25366 | 83170 | 92542 |
| LN827562_sLCL-1.19_sLCL-IM_Kenya_ND | 31203 | 36675 | 98985 | 105835 |
| LN827562_sLCL-1.19_sLCL-IM_Kenya_ND | 65110 | 68957 | 156078 | 159983 |
| LN827562_sLCL-1.19_sLCL-IM_Kenya_ND | 82531 | 85509 | 189732 | 196189 |
| LN827586_sLCL-IS1.15_sLCL-PTLD_Australia_ND | 28628 | 31067 | 95881 | 98848 |
| LN827586_sLCL-IS1.15_sLCL-PTLD_Australia_ND | 36597 | 38690 | 105756 | 112502 |
| LN827586_sLCL-IS1.15_sLCL-PTLD_Australia_ND | 42371 | 45175 | 116192 | 120807 |
| LN827586_sLCL-IS1.15_sLCL-PTLD_Australia_ND | 47509 | 56646 | 123207 | 132481 |
| LN827586_sLCL-IS1.15_sLCL-PTLD_Australia_ND | 69914 | 70937 | 160947 | 162013 |
| LN827586_sLCL-IS1.15_sLCL-PTLD_Australia_ND | 82729 | 85509 | 189930 | 196189 |
| LN827799_sLCL-IM1.16_sLCL-IM_Australia_ND | 6114 | 6777 | 6329 | 7001 |
| LN827799_sLCL-IM1.16_sLCL-IM_Australia_ND | 21556 | 22426 | 88627 | 89578 |
| LN827799_sLCL-IM1.16_sLCL-IM_Australia_ND | 51205 | 51931 | 126931 | 127670 |
| LN827799_sLCL-IM1.16_sLCL-IM_Australia_ND | 64769 | 67829 | 155737 | 158853 |
| LN827799_sLCL-IM1.16_sLCL-IM_Australia_ND | 82905 | 85509 | 192792 | 196189 |
| LN827577_sLCL-1.17_sLCL-IM_Kenya_ND | 15959 | 16499 | 82942 | 83495 |
| LN827577_sLCL-1.17_sLCL-IM_Kenya_ND | 21556 | 22642 | 88627 | 89800 |
| LN827577_sLCL-1.17_sLCL-IM_Kenya_ND | 28970 | 29893 | 96224 | 97193 |
| LN827577_sLCL-1.17_sLCL-IM_Kenya_ND | 41077 | 42625 | 114896 | 116446 |
| LN824225_HL08_HL_UK_ND | 35609 | 36172 | 103849 | 104471 |
| LN827573_sLCL-1.10_sLCL-IM_Kenya_ND | 84075 | 85509 | 193962 | 196189 |
| LN827739_LCLB95-8-del-EBER2-reseq_LCL_USA_ND | 6785 | 6788 | 7009 | 7188 |
| KR063344_RPF_BL_Brazil_1980 | 5902 | 9483 | 6116 | 62678 |
| KR063344_RPF_BL_Brazil_1980 | 42539 | 42834 | 116360 | 116655 |
| KR063344_RPF_BL_Brazil_1980 | 84652 | 85509 | 195256 | 196189 |
| LN827559_pLCL-TRL-595_sLCL_USA_ND | 9146 | 13930 | 62341 | 67194 |
| LN827559_pLCL-TRL-595_sLCL_USA_ND | 76903 | 77079 | 183855 | 184055 |
| LN827559_pLCL-TRL-595_sLCL_USA_ND | 81866 | 85509 | 188979 | 196189 |
| LN827584_sLCL-IS1.06_sLCL-IM_Australia_ND | 1 | 612 | 35 | 647 |
| LN827584_sLCL-IS1.06_sLCL-IM_Australia_ND | 84025 | 85509 | 193912 | 196189 |
| KR063345_FNR_BL_Brazil_1980 | 41912 | 42824 | 115733 | 116645 |
| KR063345_FNR_BL_Brazil_1980 | 83816 | 85509 | 193703 | 196189 |
| KP968264_H002213_BL_Argentina_1981 | 8218 | 11993 | 61383 | 65202 |
| KP968264_H002213_BL_Argentina_1981 | 21145 | 25366 | 88216 | 92542 |
| KP968264_H002213_BL_Argentina_1981 | 46312 | 47641 | 121976 | 123339 |
| KR063343_CV-ARG_BL_Argentina_1980 | 2615 | 6961 | 2712 | 7369 |
| KR063343_CV-ARG_BL_Argentina_1980 | 85348 | 85509 | 196006 | 196189 |
| KP968259_SCL_BL_Brazil_1980 | 1 | 1396 | 35 | 1433 |
| KP968259_SCL_BL_Brazil_1980 | 38040 | 39685 | 111835 | 113501 |
| KP968259_SCL_BL_Brazil_1980 | 82913 | 84110 | 192800 | 193999 |
| KT001103_SG_BL_Argentina_1981 | 5870 | 6777 | 6084 | 7001 |
| KT001103_SG_BL_Argentina_1981 | 81632 | 83339 | 188745 | 193226 |
| LN824226_HL01_HL_UK_ND | 1 | 1544 | 35 | 1581 |
| LN824226_HL01_HL_UK_ND | 9738 | 14858 | 62933 | 81766 |
| LN824226_HL01_HL_UK_ND | 26322 | 34672 | 93507 | 102652 |
| LN824226_HL01_HL_UK_ND | 39069 | 39934 | 112885 | 113750 |
| LN824226_HL01_HL_UK_ND | 42202 | 42618 | 116023 | 116439 |
| LN824226_HL01_HL_UK_ND | 46455 | 47641 | 122119 | 123339 |
| LN824226_HL01_HL_UK_ND | 82512 | 85509 | 189713 | 196189 |
| LN824204_HL05_HL_UK_ND | 36903 | 37397 | 106089 | 106583 |
| LC150338_HNNPC5_NPC_China_2013 | 1 | 2522 | 35 | 2619 |
| LC150338_HNNPC5_NPC_China_2013 | 33453 | 33526 | 101356 | 101431 |
| LC150338_HNNPC5_NPC_China_2013 | 76600 | 78680 | 183530 | 185668 |
| LC150338_HNNPC5_NPC_China_2013 | 85280 | 85509 | 195893 | 196189 |
| LN827574_sLCL-1.09_sLCL-PTLD_Kenya_ND | 1 | 107 | 35 | 141 |
| KP968257_CCH_BL_Brazil_1980 | 77578 | 79449 | 184554 | 186451 |
| KT273945_EBVaGC5_GC_China_2009 | 67085 | 68823 | 158101 | 159849 |
| KT273945_EBVaGC5_GC_China_2009 | 85322 | 85509 | 195980 | 196189 |
| KT823508_LC3_LC_China_2014 | 72197 | 72673 | 179003 | 179489 |
| LN824142_Saliva1_Health_UK_ND | 1 | 1499 | 35 | 1536 |
| LN824142_Saliva1_Health_UK_ND | 35791 | 36589 | 104090 | 105743 |
| LN824142_Saliva1_Health_UK_ND | 51206 | 51705 | 126932 | 127432 |
| KP968258_MP_BL_Brazil_1980 | 8942 | 13390 | 62136 | 66654 |
| KP968258_MP_BL_Brazil_1980 | 24959 | 25372 | 92121 | 92548 |
| KP968258_MP_BL_Brazil_1980 | 51998 | 54977 | 127737 | 130754 |
| KT273947_EBVaGC7_GC_China_2009 | 84985 | 85509 | 195592 | 196189 |
| KP968260_VGO_BL_Brazil_1980 | 1 | 13260 | 35 | 66524 |
| KP968260_VGO_BL_Brazil_1980 | 28979 | 30608 | 96233 | 98388 |
| KP968260_VGO_BL_Brazil_1980 | 33857 | 37411 | 101800 | 106597 |
| KP968260_VGO_BL_Brazil_1980 | 40513 | 42944 | 114329 | 116766 |
| KP968260_VGO_BL_Brazil_1980 | 60904 | 62169 | 137900 | 139170 |
| KP968260_VGO_BL_Brazil_1980 | 65306 | 66354 | 156274 | 157369 |
| KP968260_VGO_BL_Brazil_1980 | 77875 | 80498 | 184851 | 187570 |
| KP968260_VGO_BL_Brazil_1980 | 84064 | 85509 | 193951 | 196189 |
| KT273948_EBVaGC8_GC_China_2010 | 8776 | 12108 | 61959 | 65347 |
| KT273948_EBVaGC8_GC_China_2010 | 29432 | 31067 | 96702 | 98848 |
| KT273948_EBVaGC8_GC_China_2010 | 47273 | 47641 | 122971 | 123339 |
| KT273948_EBVaGC8_GC_China_2010 | 51793 | 57553 | 127520 | 133396 |
| KT273944_EBVaGC4_GC_China_2014 | 30871 | 31075 | 98651 | 98856 |
| KT273944_EBVaGC4_GC_China_2014 | 43353 | 46557 | 117179 | 122221 |
| AY961628_GD1_NPC-saliva_China_ND | 2966 | 4428 | 3063 | 4587 |
| AY961628_GD1_NPC-saliva_China_ND | 38040 | 42944 | 111835 | 116766 |
| AY961628_GD1_NPC-saliva_China_ND | 69756 | 71286 | 160786 | 178092 |
| KT823507_LC2_LC_China_2014 | 1119 | 1159 | 1156 | 1196 |
| LC149491_HNNPC2_NPC_China_2013 | 58362 | 58777 | 135231 | 135732 |
| LC149491_HNNPC2_NPC_China_2013 | 65684 | 69214 | 156652 | 160241 |
| LC149491_HNNPC2_NPC_China_2013 | 84326 | 85509 | 194408 | 196189 |
| KT273946_EBVaGC6_GC_China_2009 | 1 | 3695 | 35 | 3840 |
| KT273946_EBVaGC6_GC_China_2009 | 6860 | 13977 | 7268 | 67241 |
| KT273946_EBVaGC6_GC_China_2009 | 22363 | 26283 | 89514 | 93468 |
| KT273946_EBVaGC6_GC_China_2009 | 39524 | 43097 | 113340 | 116919 |
| KT273946_EBVaGC6_GC_China_2009 | 51205 | 52223 | 126931 | 127964 |
| KT273946_EBVaGC6_GC_China_2009 | 82960 | 85509 | 192847 | 196189 |
| KT823509_LC4_LC_China_2014 | 6792 | 6977 | 7192 | 7386 |
| KT823509_LC4_LC_China_2014 | 15681 | 24047 | 82648 | 91208 |
| KT823509_LC4_LC_China_2014 | 71558 | 79228 | 178364 | 186217 |
| LN827523_L591_Hl-cell-line_Germany_ND | 81476 | 85509 | 188577 | 196189 |
| KF992568_HKNPC6_NPC-tumor_China_2008 | 30775 | 31123 | 98555 | 98905 |
| KF992568_HKNPC6_NPC-tumor_China_2008 | 84277 | 85509 | 194290 | 196189 |
| LC137018_HNNPC1_NPC-tumor_China_2013 | 85369 | 85509 | 196046 | 196189 |
| KF992567_HKNPC5_NPC-tumor_China_2008 | 84664 | 85509 | 195268 | 196189 |
| LC150742_HNNPC7_NPC-tumor_China_2013 | 42284 | 43208 | 116105 | 117030 |
| LC150742_HNNPC7_NPC-tumor_China_2013 | 46002 | 48661 | 121641 | 124382 |
| LC150742_HNNPC7_NPC-tumor_China_2013 | 84382 | 85509 | 194986 | 196189 |
| KP735248_GC1_GC-cell-line_SouthKorea_2010 | 85310 | 85509 | 195923 | 196189 |
| LC150743_HNNPC8_NPC-tumor_China_2013 | 84877 | 85509 | 195484 | 196189 |
| KX125050_GC-EBV1_GC_China_2011 | 37590 | 41527 | 110796 | 115347 |
| KF717093_Raji_BL_Nigeria_ND | 10062 | 23875 | 63257 | 91036 |
| KF717093_Raji_BL_Nigeria_ND | 30851 | 34009 | 98631 | 101982 |
| KF717093_Raji_BL_Nigeria_ND | 36247 | 37313 | 105400 | 106499 |
| KF717093_Raji_BL_Nigeria_ND | 46847 | 50369 | 122511 | 126091 |
| KF717093_Raji_BL_Nigeria_ND | 82905 | 84826 | 192792 | 195433 |
| MF547454_E1583_OWv1_IM_USA_2013 | 29176 | 30871 | 96446 | 98651 |
| MF547456_E1583_OWv7_IM_USA_2014 | 42820 | 43403 | 116641 | 117229 |
| MF547465_E1536_BCv7_IM_USA_2013 | 42371 | 44007 | 116192 | 119561 |
| MF547465_E1536_BCv7_IM_USA_2013 | 56224 | 56235 | 132059 | 132070 |
| MF547465_E1536_BCv7_IM_USA_2013 | 84256 | 85509 | 194269 | 196189 |
| MF547467_E1548_OWv1_IM_USA_2013 | 74982 | 74990 | 181866 | 181874 |
| MF547467_E1548_OWv1_IM_USA_2013 | 84306 | 85509 | 194373 | 196189 |
| MF547469_E1548_OWv7_IM_USA_2013 | 74978 | 75236 | 181862 | 182122 |
| MF547471_E1563_OWv7_IM_USA_2014 | 83750 | 84444 | 193637 | 195048 |
| MF547472_E1563_BCV7_IM_USA_2014 | 37859 | 39101 | 111072 | 112917 |
| MF547472_E1563_BCV7_IM_USA_2014 | 60769 | 62052 | 137765 | 139053 |
| MF547472_E1563_BCV7_IM_USA_2014 | 77010 | 79030 | 183962 | 186019 |
| MF547472_E1563_BCV7_IM_USA_2014 | 84256 | 85509 | 194269 | 196189 |
| MF547474_E1590_OWv1_IM_USA_2014 | 29782 | 30308 | 97052 | 98088 |
| MF547476_E1590_OWv7_IM_USA_2014 | 6717 | 7438 | 6941 | 60602 |
| MF547479_E1492_BCv7_IM_USA_2011 | 82960 | 84112 | 192847 | 194001 |
| MF547485_E1578_BCv1_IM_USA_2013 | 84668 | 85509 | 195272 | 196189 |
| MF547486_E1578_BCv7_IM_USA_2014 | 11223 | 12108 | 64428 | 65347 |
| MF547486_E1578_BCv7_IM_USA_2014 | 42771 | 44131 | 116592 | 119685 |
| MF547486_E1578_BCv7_IM_USA_2014 | 84328 | 85509 | 194410 | 196189 |
| MF547487_E1578_OWv7_IM_USA_2014 | 60769 | 61198 | 137765 | 138196 |
| MF547487_E1578_OWv7_IM_USA_2014 | 84766 | 85509 | 195370 | 196189 |
| MF547488_E1578_OWv1_IM_USA_2013 | 22130 | 22228 | 89204 | 89302 |
| MF547489_E1577_BCv1_IM_USA_2013 | 84289 | 84293 | 194356 | 194360 |
| MF547490_E1577_OWv1_IM_USA_2013 | 42816 | 43869 | 116637 | 119423 |
| MF547491_E1577_OWv7_IM_USA_ND | 83620 | 84543 | 193507 | 195147 |
| MF547492_E1577_BCv7_IM_USA_ND | 6719 | 7736 | 6943 | 60901 |
| MF547492_E1577_BCv7_IM_USA_ND | 35822 | 36787 | 104121 | 105955 |
| MF547492_E1577_BCv7_IM_USA_ND | 83990 | 85509 | 193877 | 196189 |
| MG021317_EBVaGC5-1_GC_USA_2013 | 22365 | 23194 | 89517 | 90352 |
| MG021317_EBVaGC5-1_GC_USA_2013 | 37590 | 39758 | 110796 | 113574 |
| MG021317_EBVaGC5-1_GC_USA_2013 | 42430 | 42944 | 116251 | 116766 |
| MG021317_EBVaGC5-1_GC_USA_2013 | 45124 | 47641 | 120756 | 123339 |
| MG021317_EBVaGC5-1_GC_USA_2013 | 67085 | 68823 | 158101 | 159849 |
| MG021308_Mutu-GC1_GC_USA_2013 | 15594 | 16209 | 82560 | 83197 |
| MG021307_Akata-GC1_GC_USA_2013 | 42449 | 43208 | 116270 | 117030 |
| MG021307_Akata-GC1_GC_USA_2013 | 83913 | 85509 | 193800 | 196189 |
| MG021312_Akata-GC1_GC_USA_2017 | 1 | 1098 | 35 | 1135 |
| MG021316_HKNPC6-GC1_GC_Vietnam_2013 | 1 | 1160 | 35 | 1197 |
| MG021316_HKNPC6-GC1_GC_Vietnam_2013 | 18612 | 26534 | 85667 | 93719 |
| MG021316_HKNPC6-GC1_GC_Vietnam_2013 | 29241 | 36444 | 96511 | 105597 |
| MG021316_HKNPC6-GC1_GC_Vietnam_2013 | 39374 | 39779 | 113190 | 113595 |
| MG021316_HKNPC6-GC1_GC_Vietnam_2013 | 52037 | 59197 | 127778 | 136188 |
| MG021316_HKNPC6-GC1_GC_Vietnam_2013 | 65474 | 67085 | 156442 | 158101 |
| MG021316_HKNPC6-GC1_GC_Vietnam_2013 | 75523 | 82693 | 182418 | 189894 |
| MG021310_Mutu-GC3_GC_USA_2013 | 84555 | 85509 | 195159 | 196189 |
| MG021311_Mutu-GC4_GC_USA_2013 | 83222 | 85509 | 193109 | 196189 |
| MG021309_Mutu-GC2_GC_USA_2013 | 46987 | 47662 | 122685 | 123360 |
| MG021309_Mutu-GC2_GC_USA_2013 | 84025 | 85509 | 193912 | 196189 |
| NODE_190 | 76903 | 79078 | 183855 | 186067 |
| NODE_190 | 84167 | 85509 | 194180 | 196189 |
| NODE_191 | 74902 | 76112 | 181786 | 183042 |
| NODE_191 | 81476 | 82753 | 188577 | 189954 |
| NODE_192 | 84326 | 85509 | 194408 | 196189 |
| NODE_208 | 7438 | 13351 | 60602 | 66615 |
| NODE_209 | 46455 | 46988 | 122119 | 122686 |
| NODE_209 | 63431 | 64359 | 154388 | 155324 |
| NODE_210 | 5687 | 6977 | 5898 | 7386 |
| NODE_210 | 54834 | 54977 | 130611 | 130754 |
| NODE_210 | 60845 | 62052 | 137841 | 139053 |
| NODE_210 | 71256 | 72210 | 178062 | 179016 |
| NODE_214 | 5944 | 6389 | 6158 | 6613 |
| NODE_214 | 14080 | 17692 | 80733 | 84696 |
| NODE_214 | 21391 | 22495 | 88462 | 89651 |
| NODE_214 | 42539 | 45166 | 116360 | 120798 |
| NODE_214 | 83837 | 85509 | 193724 | 196189 |
| NODE_215 | 10934 | 12324 | 64139 | 65563 |
| NODE_215 | 21292 | 22612 | 88363 | 89770 |
| NODE_215 | 42809 | 43804 | 116630 | 119358 |
| NODE_215 | 54123 | 54927 | 129879 | 130704 |
| NODE_215 | 84332 | 85509 | 194414 | 196189 |
| NODE_216 | 54874 | 55344 | 130651 | 131153 |
| NODE_216 | 82729 | 85509 | 189930 | 196189 |
| NODE_217 | 53556 | 56553 | 129311 | 132388 |
| NODE_217 | 65306 | 68957 | 156274 | 159983 |
| NODE_217 | 74126 | 76112 | 181010 | 183042 |
| NODE_217 | 80187 | 82693 | 187252 | 189894 |
| NODE_218 | 39524 | 39779 | 113340 | 113595 |
| NODE_218 | 42255 | 42801 | 116076 | 116622 |
| NODE_218 | 46505 | 46988 | 122169 | 122686 |
| NODE_219 | 10062 | 10755 | 63257 | 63960 |
| NODE_219 | 60769 | 62043 | 137765 | 139044 |
| NODE_219 | 82960 | 85509 | 192847 | 196189 |
| NODE_220 | 33677 | 39405 | 101619 | 113221 |
| NODE_221 | 33888 | 37411 | 101831 | 106597 |
| NODE_221 | 42595 | 42780 | 116416 | 116601 |
| NODE_221 | 71256 | 71830 | 178062 | 178636 |
| NODE_222 | 33381 | 35487 | 101239 | 103481 |
| NODE_222 | 44875 | 46524 | 120496 | 122188 |
| NODE_224 | 42255 | 43463 | 116076 | 117289 |
| NODE_224 | 71256 | 72673 | 178062 | 179489 |
| NODE_224 | 76273 | 82965 | 183203 | 192852 |
| NODE_225 | 3006 | 3413 | 3112 | 3558 |
| NODE_226 | 1 | 1957 | 35 | 1995 |
| NODE_227 | 63431 | 64359 | 154388 | 155324 |
| NODE_227 | 84490 | 85509 | 195094 | 196189 |
| NODE_228 | 46452 | 51705 | 122116 | 127432 |
| NODE_228 | 54814 | 54977 | 130591 | 130754 |
| NODE_228 | 60629 | 62169 | 137625 | 139170 |
| NODE_229 | 1 | 472 | 35 | 506 |
| NODE_229 | 76273 | 77266 | 183203 | 184242 |
| NODE_238 | 44576 | 47641 | 120197 | 123339 |
| NODE_239 | 39710 | 42374 | 113526 | 116195 |
| NODE_239 | 50958 | 56389 | 126683 | 132224 |
| NODE_240 | 1 | 1159 | 35 | 1196 |
| NODE_240 | 14170 | 21674 | 80957 | 88745 |
| NODE_240 | 75430 | 76112 | 182325 | 183042 |
| NODE_241 | 18532 | 30050 | 85575 | 97830 |
| NODE_241 | 49426 | 51225 | 125147 | 126951 |
| NODE_242 | 11020 | 11895 | 64225 | 65104 |
| NODE_242 | 30851 | 37949 | 98631 | 111162 |
| NODE_242 | 42539 | 42689 | 116360 | 116510 |
| NODE_243 | 14400 | 20058 | 81188 | 87129 |
| NODE_243 | 38040 | 39614 | 111835 | 113430 |
| NODE_244 | 38068 | 39685 | 111863 | 113501 |
| NODE_244 | 47033 | 49186 | 122731 | 124907 |
| NODE_244 | 79171 | 82693 | 186160 | 189894 |
| NODE_245 | 36132 | 38305 | 104431 | 112113 |
| NODE_245 | 46452 | 46848 | 122116 | 122512 |
| NODE_245 | 72424 | 73481 | 179230 | 180332 |
| NODE_246 | 1 | 1356 | 35 | 1393 |
| NODE_246 | 5902 | 6977 | 6116 | 7386 |
| NODE_246 | 39661 | 39779 | 113477 | 113595 |
| NODE_246 | 81476 | 82693 | 188577 | 189894 |
| NODE_247 | 1 | 1879 | 35 | 1917 |
| NODE_247 | 12707 | 15121 | 65946 | 82076 |
| NODE_247 | 20451 | 22612 | 87522 | 89770 |
| NODE_247 | 41803 | 45175 | 115624 | 120807 |
| NODE_247 | 63923 | 69356 | 154886 | 160385 |
| NODE_247 | 79161 | 85509 | 186150 | 196189 |
| NODE_248 | 29782 | 37660 | 97052 | 110866 |
| NODE_248 | 67085 | 68957 | 158101 | 159983 |
| NODE_250 | 1 | 1160 | 35 | 1197 |
| NODE_250 | 33888 | 35639 | 101831 | 103879 |
| NODE_250 | 37786 | 38690 | 110992 | 112502 |
| NODE_250 | 65175 | 66597 | 156143 | 157612 |
| NODE_251 | 34047 | 37411 | 102020 | 106597 |
| NODE_252 | 35618 | 36115 | 103858 | 104414 |
| NODE_252 | 42539 | 42780 | 116360 | 116601 |
| NODE_252 | 48652 | 50801 | 124373 | 126523 |
| NODE_252 | 82648 | 82965 | 189849 | 192852 |
| NODE_253 | 37786 | 43463 | 110992 | 117289 |
| NODE_257 | 61862 | 63431 | 138863 | 154388 |
| NODE_257 | 66198 | 66597 | 157213 | 157612 |
| NODE_259 | 42365 | 42479 | 116186 | 116300 |
| NODE_262 | 83339 | 85509 | 193226 | 196189 |
| NODE_263 | 43643 | 46050 | 119197 | 121689 |
| NODE_263 | 57301 | 58143 | 133144 | 134844 |
| NODE_264 | 14497 | 15085 | 81395 | 82040 |
| NODE_264 | 42771 | 43463 | 116592 | 117289 |
| NODE_264 | 82828 | 85509 | 192715 | 196189 |
| NODE_265 | 76528 | 79449 | 183458 | 186451 |
| NODE_265 | 81632 | 82753 | 188745 | 189954 |
| NODE_266 | 1 | 1396 | 35 | 1433 |
| NODE_266 | 42539 | 44444 | 116360 | 120065 |
| NODE_267 | 15347 | 16665 | 82311 | 83661 |
| NODE_267 | 33355 | 37542 | 101213 | 106728 |
| NODE_268 | 21145 | 22612 | 88216 | 89770 |
| NODE_269 | 36601 | 37500 | 105760 | 106686 |
| NODE_269 | 82960 | 85509 | 192847 | 196189 |
| NODE_270 | 57301 | 60845 | 133144 | 137841 |
| NODE_271 | 34753 | 42263 | 102733 | 116084 |
| NODE_273 | 38913 | 39890 | 112726 | 113706 |
| NODE_273 | 45681 | 46859 | 121313 | 122523 |
| NODE_273 | 54401 | 55344 | 130157 | 131153 |
| NODE_274 | 78238 | 80415 | 185214 | 187487 |
| NODE_275 | 32977 | 38690 | 100795 | 112502 |
| NODE_276 | 76528 | 77266 | 183458 | 184242 |
| NODE_276 | 81506 | 82753 | 188608 | 189954 |
| NODE_278 | 9206 | 10182 | 62401 | 63387 |
| NODE_278 | 34876 | 36138 | 102856 | 104437 |
| NODE_278 | 81506 | 85509 | 188608 | 196189 |
| NODE_279 | 33468 | 38346 | 101371 | 112154 |
| NODE_279 | 44875 | 45175 | 120496 | 120807 |
| NODE_280 | 49786 | 56389 | 125507 | 132224 |
| NODE_281 | 1 | 1544 | 35 | 1581 |
| NODE_281 | 28665 | 30096 | 95919 | 97876 |
| NODE_281 | 83815 | 84110 | 193702 | 193999 |
| NODE_282 | 3866 | 6717 | 4011 | 6941 |
| NODE_282 | 28979 | 34324 | 96233 | 102301 |
| NODE_282 | 37313 | 40457 | 106499 | 114273 |
| NODE_283 | 82960 | 84328 | 192847 | 194410 |
| NODE_284 | 60708 | 62163 | 137704 | 139164 |
| NODE_285 | 11786 | 12736 | 64992 | 65975 |
| NODE_285 | 28859 | 34545 | 96113 | 102525 |
| NODE_286 | 21307 | 21736 | 88378 | 88807 |
| NODE_286 | 33878 | 36339 | 101821 | 105492 |
| NODE_290 | 29782 | 30267 | 97052 | 98047 |
| NODE_294 | 82825 | 83345 | 192712 | 193232 |
| NODE_295 | 10835 | 11664 | 64040 | 64870 |
| NODE_295 | 48486 | 49078 | 124185 | 124799 |
| NODE_295 | 51463 | 52019 | 127190 | 127758 |
| NODE_295 | 84295 | 85509 | 194362 | 196189 |
| NODE_296 | 60484 | 62043 | 137480 | 139044 |
| NODE_297 | 42539 | 44191 | 116360 | 119745 |
| NODE_299 | 83339 | 85509 | 193226 | 196189 |
| NODE_300 | 13104 | 17131 | 66367 | 84132 |
| NODE_300 | 33736 | 34190 | 101678 | 102163 |
| NODE_300 | 47324 | 48268 | 123022 | 123967 |
| NODE_300 | 54874 | 56432 | 130651 | 132267 |
| NODE_301 | 42795 | 43097 | 116616 | 116919 |
| NODE_301 | 46847 | 49827 | 122511 | 125548 |
| NODE_302 | 34876 | 37137 | 102856 | 106323 |
| NODE_302 | 42737 | 43463 | 116558 | 117289 |
| NODE_303 | 21961 | 22607 | 89032 | 89765 |
| NODE_303 | 41803 | 43097 | 115624 | 116919 |
| NODE_304 | 82905 | 85509 | 192792 | 196189 |
| NODE_305 | 42371 | 42689 | 116192 | 116510 |
| NODE_305 | 49897 | 54784 | 125618 | 130558 |
| NODE_305 | 75301 | 85509 | 182196 | 196189 |
| NODE_306 | 15121 | 16499 | 82076 | 83495 |
| NODE_306 | 33878 | 35709 | 101821 | 103958 |
| NODE_306 | 84328 | 85509 | 194410 | 196189 |
| NODE_308 | 84108 | 85509 | 193997 | 196189 |
| NODE_309 | 22165 | 22607 | 89239 | 89765 |
| NODE_309 | 60769 | 62043 | 137765 | 139044 |
| NODE_309 | 63713 | 64359 | 154670 | 155324 |
| NODE_312 | 5870 | 9375 | 6084 | 62570 |
| NODE_312 | 82905 | 84005 | 192792 | 193892 |
| NODE_313 | 3866 | 4428 | 4011 | 4587 |
| NODE_313 | 32759 | 33526 | 100550 | 101431 |
| NODE_313 | 42371 | 43374 | 116192 | 117200 |
| NODE_313 | 49066 | 51998 | 124787 | 127737 |
| NODE_323 | 75639 | 80498 | 182544 | 187570 |
| NODE_323 | 82901 | 84112 | 192788 | 194001 |
| NODE_324 | 28970 | 30096 | 96224 | 97876 |
| NODE_324 | 43310 | 44194 | 117136 | 119748 |
| NODE_324 | 47033 | 48232 | 122731 | 123931 |
| NODE_324 | 54874 | 56432 | 130651 | 132267 |
| NODE_324 | 82905 | 82965 | 192792 | 192852 |
| NODE_325 | 1 | 1396 | 35 | 1433 |
| NODE_325 | 29925 | 30578 | 97705 | 98358 |
| NODE_325 | 71256 | 71738 | 178062 | 178544 |
| NODE_325 | 84679 | 85509 | 195283 | 196189 |
| NODE_326 | 61090 | 68957 | 138088 | 159983 |
| NODE_327 | 12714 | 13371 | 65953 | 66635 |
| NODE_327 | 36787 | 37852 | 105955 | 111065 |
| NODE_327 | 51765 | 52360 | 127492 | 128102 |
| NODE_327 | 84108 | 84679 | 193997 | 195283 |
| NODE_328 | 84326 | 85509 | 194408 | 196189 |
| NODE_329 | 16167 | 16481 | 83155 | 83477 |
| NODE_335 | 72670 | 73715 | 179486 | 180599 |
| NODE_335 | 84269 | 84444 | 194282 | 195048 |
| NODE_342 | 84326 | 85509 | 194408 | 196189 |
| NODE_345 | 84328 | 85509 | 194410 | 196189 |
| NODE_346 | 28665 | 30578 | 95919 | 98358 |
| NODE_346 | 84025 | 84083 | 193912 | 193970 |
| NODE_348 | 6089 | 6849 | 6304 | 7256 |
| NODE_348 | 39405 | 39717 | 113221 | 113533 |
| NODE_349 | 84025 | 84800 | 193912 | 195407 |
| NODE_350 | 33525 | 33892 | 101430 | 101835 |
| NODE_350 | 39083 | 40833 | 112899 | 114652 |
| NODE_351 | 39589 | 39717 | 113405 | 113533 |
| NODE_352 | 11020 | 11664 | 64225 | 64870 |
| NODE_352 | 77010 | 77086 | 183962 | 184062 |
| NODE_354 | 42422 | 44194 | 116243 | 119748 |
| NODE_354 | 82905 | 84679 | 192792 | 195283 |
| NODE_355 | 42903 | 44191 | 116725 | 119745 |
| NODE_357 | 85391 | 85509 | 196068 | 196189 |
| NODE_359 | 82903 | 83245 | 192790 | 193132 |
| NODE_359 | 85145 | 85509 | 195756 | 196189 |
| NODE_364 | 70044 | 71076 | 161082 | 162169 |
| NODE_366 | 47273 | 47525 | 122971 | 123223 |
| NODE_367 | 70446 | 71080 | 161496 | 162173 |

**Supplementary table S6.** Quantitative results of mutations and recombination events from the genome dataset obtained by Gubbins. This analysis was performed on whole genome alignment after the Gblocks processing (a final alignment length of 85509 bp).

| Node | Total SNPs | Num of SNPs inside recombinations | Num of SNPs outside recombinations | Num of Recombination Blocks | Bases in Recombinations | r/m | rho/theta | Bases in Clonal Frame |
| --- | --- | --- | --- | --- | --- | --- | --- | --- |
| NC_007605_B95.8Raji_IM_USA_ND | 0 | 0 | 0 | 0 | 0 | 0 | 0 | 85509 |
| NC_009334_AG876_BL_Ghana_ND | 0 | 0 | 0 | 0 | 20589 | 0 | 0 | 64916 |
| AP015016_YCCEL1_GC-cell-line_Korea_ND | 0 | 0 | 0 | 0 | 31626 | 0 | 0 | 53880 |
| KF373730_M81_NPC_China_1970 | 14 | 0 | 14 | 0 | 39668 | 0 | 0 | 45833 |
| LN827556_Cheptages_BL_Kenya_ND | 144 | 0 | 144 | 0 | 10413 | 0 | 0 | 75094 |
| LN827576_sLCL-IS1.20_sLCL-IM_Australia_ND | 100 | 44 | 56 | 1 | 13484 | 0.785714 | 0.017857 | 77021 |
| LN827580_sLCL-2.16_sLCL-IM_Kenya_ND | 154 | 28 | 126 | 1 | 14048 | 0.222222 | 0.007937 | 75094 |
| LN827596_sLCL-IM1.02_sLCL-IM_Australia_ND | 17 | 0 | 17 | 0 | 20039 | 0 | 0 | 65467 |
| LN827560_sLCL-2.14_sLCL-IM_Kenya_ND | 0 | 0 | 0 | 0 | 20589 | 0 | 0 | 64916 |
| LN827587_sLCL-2.21_sLCL-IM_Kenya_ND | 0 | 0 | 0 | 0 | 20589 | 0 | 0 | 64916 |
| LN827578_sLCL-IS1.13_sLCL-IM_Australia_ND | 135 | 124 | 11 | 2 | 21123 | 11.272727 | 0.181818 | 69812 |
| LN827800_Jijoy_BL_Nigeria_ND | 1 | 0 | 1 | 0 | 10413 | 0 | 0 | 75094 |
| LN827575_sLCL-IS1.14_sLCL-IM_Australia_ND | 9 | 0 | 9 | 0 | 8486 | 0 | 0 | 77021 |
| LN827591_sLCL-2.15_sLCL-PTLD_Kenya_ND | 46 | 35 | 11 | 1 | 33379 | 3.181818 | 0.090909 | 60874 |
| LN827590_sLCL-IM1.05_sLCL-IM_Australia_ND | 15 | 0 | 15 | 0 | 15694 | 0 | 0 | 69812 |
| LN831023_sLCL-2.22_sLCL-PTLD_Kenya_ND | 0 | 0 | 0 | 0 | 20589 | 0 | 0 | 64916 |
| LN827545_Daudi_BL_Kenya_ND | 184 | 0 | 184 | 0 | 4478 | 0 | 0 | 81028 |
| LN827548_P3HR1c16_BL_Nigeria_ND | 0 | 0 | 0 | 0 | 10413 | 0 | 0 | 75094 |
| LN827567_sLCL-IM1.09_sLCL-IM_Australia_ND | 94 | 0 | 94 | 0 | 8486 | 0 | 0 | 77021 |
| LN827583_sLCL-IM1.17_sLCL-IM_Australia_ND | 14 | 0 | 14 | 0 | 8486 | 0 | 0 | 77021 |
| LN827589_sLCL-IS2.01_sLCL-IM_Australia_ND | 682 | 589 | 93 | 4 | 17736 | 6.333333 | 0.043011 | 70799 |
| LN824209_HKN14_sLCB_China_ND | 184 | 26 | 158 | 1 | 27112 | 0.164557 | 0.006329 | 61129 |
| LN827588_SLCL-IS1.19_sLCL-IM_Australia_ND | 20 | 0 | 20 | 0 | 8486 | 0 | 0 | 77021 |
| LN827524_HL11_HL_UK_ND | 33 | 0 | 33 | 0 | 2079 | 0 | 0 | 83428 |
| LN827544_Wewak1_BL_PapuaNewGuinea_ND | 958 | 564 | 394 | 3 | 15430 | 1.431472 | 0.007614 | 70799 |
| LN827557_Bl36_BL_NorthAfrica_ND | 20 | 20 | 0 | 1 | 14644 | 0 | 0 | 75094 |
| LN827547_HKN15_sLCB_China_ND | 0 | 0 | 0 | 0 | 39412 | 0 | 0 | 46091 |
| LN824224_HKN19_sLCB_China_ND | 0 | 0 | 0 | 0 | 39412 | 0 | 0 | 46091 |
| LN827570_sLCL-IS1.01_sLCL-PTLD_Australia_ND | 11 | 0 | 11 | 0 | 10640 | 0 | 0 | 74867 |
| LN827569_sLCL-IS1.11_sLCL-PTLD_Australia_ND | 7 | 0 | 7 | 0 | 3904 | 0 | 0 | 81602 |
| LN827592_sLCL-IS1.10_sLCL-PTLD_Australia_ND | 27 | 0 | 27 | 0 | 3904 | 0 | 0 | 81602 |
| LN827572_sLCL-IS1.18_sLCL-PTLD_Australia_ND | 13 | 0 | 13 | 0 | 3722 | 0 | 0 | 81786 |
| LN827593_sLCL-IS1.12_sLCL-PTLD_Australia_ND | 13 | 0 | 13 | 0 | 8486 | 0 | 0 | 77021 |
| KT273942_EBVaGC1_GC_China_2013 | 130 | 39 | 91 | 1 | 31626 | 0.428571 | 0.010989 | 53880 |
| LN827527_M-ABA_LCL-NFC_NorthAfrica_ND | 38 | 16 | 22 | 1 | 13777 | 0.727273 | 0.045455 | 77021 |
| LN827564_HL04_HL_UK_ND | 17 | 0 | 17 | 0 | 15694 | 0 | 0 | 69812 |
| LN824205_sLCL-1.12_sLCL-PTLD_Australia_ND | 235 | 100 | 135 | 2 | 12136 | 0.740741 | 0.014815 | 81028 |
| LN827594_sLCL-IS1.0.7_sLCL-PTLD_Australia_ND | 47 | 29 | 18 | 1 | 44323 | 1.611111 | 0.055556 | 41181 |
| LN827522_HL09_HL_UK_ND | 9 | 0 | 9 | 0 | 20039 | 0 | 0 | 65467 |
| LN827549_D3201.2_NPC-biopsy_China_ND | 11 | 0 | 11 | 0 | 29128 | 0 | 0 | 56376 |
| LN827561_YCCEL1_GC-cell-line_SouthKorea_ND | 0 | 0 | 0 | 0 | 31626 | 0 | 0 | 53880 |
| LN827582_sLCL-BL1.03_sLCL-PTLD_Kenya_ND | 136 | 125 | 11 | 3 | 20841 | 11.363636 | 0.272727 | 80855 |
| LN827526_Bl37_BL_Africa_ND | 80 | 53 | 27 | 2 | 29821 | 1.962963 | 0.074074 | 67154 |
| LN827562_sLCL-1.19_sLCL-IM_Kenya_ND | 373 | 145 | 228 | 3 | 16216 | 0.635965 | 0.013158 | 70799 |
| LN827586_sLCL-IS1.15_sLCL-PTLD_Australia_ND | 220 | 90 | 130 | 4 | 17117 | 0.692308 | 0.030769 | 70799 |
| LN827525_C666-1-reseq_NPC-cell-line_China_ND | 0 | 0 | 0 | 0 | 39668 | 0 | 0 | 45833 |
| LN827581_sLCL-IM1.05_sLCL-IM_Kenya_ND | 11 | 0 | 11 | 0 | 44323 | 0 | 0 | 41181 |
| LN827550_sLCL-1.11_sLCL-IM_Kenya_ND | 6 | 0 | 6 | 0 | 4650 | 0 | 0 | 80855 |
| LN827563_sLCL-1.18_sLCL-IM_Kenya_ND | 7 | 0 | 7 | 0 | 24630 | 0 | 0 | 60874 |
| LN827566_sLCL-1.06_sLCL-IM_Kenya_ND | 5 | 0 | 5 | 0 | 4650 | 0 | 0 | 80855 |
| LN827571_sLCL-BL1.20_sLCL-PTLD_Kenya_ND | 5 | 0 | 5 | 0 | 4650 | 0 | 0 | 80855 |
| LN827585_sLCL-1.04_sLCL-IM_Kenya_ND | 61 | 33 | 28 | 1 | 12523 | 1.178571 | 0.035714 | 80855 |
| LN827799_sLCL-IM1.16_sLCL-IM_Australia_ND | 281 | 138 | 143 | 3 | 24268 | 0.965035 | 0.020979 | 64299 |
| LN827577_sLCL-1.17_sLCL-IM_Kenya_ND | 151 | 0 | 151 | 0 | 4650 | 0 | 0 | 80855 |
| LN827552_sLCL-1.08_sLCL-IM_Kenya_ND | 13 | 0 | 13 | 0 | 4650 | 0 | 0 | 80855 |
| LN827568_sLCL-1.24_sLCL-IM_Kenya_ND | 15 | 0 | 15 | 0 | 44323 | 0 | 0 | 41181 |
| LN827546_HL02_HL_UK_ND | 8 | 0 | 8 | 0 | 10640 | 0 | 0 | 74867 |
| LN824225_HL08_HL_UK_ND | 18 | 15 | 3 | 1 | 15216 | 5 | 0.333333 | 74867 |
| LN827573_sLCL-1.10_sLCL-IM_Kenya_ND | 29 | 17 | 12 | 1 | 5690 | 1.416667 | 0.083333 | 80855 |
| LN827739_LCLB95-8-del-EBER2-reseq_LCL_USA_ND | 5 | 0 | 5 | 0 | 0 | 0 | 0 | 85509 |
| LN827565_sLCL-1.07_sLCL-IM_Kenya_ND | 7 | 0 | 7 | 0 | 4650 | 0 | 0 | 80855 |
| KR063344_RPF_BL_Brazil_1980 | 232 | 74 | 158 | 1 | 19803 | 0.468354 | 0.006329 | 69283 |
| LN827558_sLCL-1.02_sLCL-IM_Kenya_ND | 11 | 0 | 11 | 0 | 4650 | 0 | 0 | 80855 |
| LN827553_sLCL-IS1.08_sLCL-IM_Australia_ND | 24 | 0 | 24 | 0 | 3904 | 0 | 0 | 81602 |
| KT273949_EBVaGC9_GC_China_2010 | 16 | 0 | 16 | 0 | 35550 | 0 | 0 | 49955 |
| LN827559_pLCL-TRL-595_sLCL_USA_ND | 147 | 102 | 45 | 3 | 21226 | 2.266667 | 0.066667 | 75829 |
| KC440852_K4413-Mi_sLCL_USA_2012 | 16 | 0 | 16 | 0 | 8486 | 0 | 0 | 77021 |
| LN824203_Mak1-duplicate_BL_Kenya_ND | 0 | 0 | 0 | 0 | 20166 | 0 | 0 | 65337 |
| LN827584_sLCL-IS1.06_sLCL-IM_Australia_ND | 54 | 29 | 25 | 2 | 14266 | 1.16 | 0.08 | 75829 |
| KR063345_FNR_BL_Brazil_1980 | 164 | 28 | 136 | 1 | 3231 | 0.205882 | 0.007353 | 83295 |
| KR063342_H03753A_BL_Argentina_1981 | 2 | 0 | 2 | 0 | 26190 | 0 | 0 | 59313 |
| KP968264_H002213_BL_Argentina_1981 | 68 | 0 | 68 | 0 | 30703 | 0 | 0 | 54800 |
| AJ507799_B95.8Raji_IM_USA_ND | 0 | 0 | 0 | 0 | 0 | 0 | 0 | 85509 |
| KP968263_H058015C_BL_Argentina_1990 | 0 | 0 | 0 | 0 | 24360 | 0 | 0 | 61144 |
| KR063343_CV-ARG_BL_Argentina_1980 | 123 | 114 | 9 | 2 | 52422 | 12.666667 | 0.222222 | 41181 |
| KP968259_SCL_BL_Brazil_1980 | 140 | 67 | 73 | 2 | 27702 | 0.917808 | 0.027397 | 60428 |
| LN827555_X50-7_LCL_USA_ND | 3 | 0 | 3 | 0 | 0 | 0 | 0 | 85509 |
| KT001103_SG_BL_Argentina_1981 | 57 | 23 | 34 | 1 | 33467 | 0.676471 | 0.029412 | 53443 |
| KP968261_HU11393_BL_Ghana_1980 | 0 | 0 | 0 | 0 | 26190 | 0 | 0 | 59313 |
| LN824226_HL01_HL_UK_ND | 288 | 60 | 228 | 2 | 14707 | 0.263158 | 0.008772 | 70799 |
| KP968262_H018436D_BL_Argentina_1976 | 1 | 0 | 1 | 0 | 24360 | 0 | 0 | 61144 |
| KC440851_K4123-Mi_sLCL_USA_2012 | 41 | 0 | 41 | 0 | 44323 | 0 | 0 | 41181 |
| LN827579_sLCL-1.13_sLCL-IM_Kenya_ND | 6 | 0 | 6 | 0 | 44323 | 0 | 0 | 41181 |
| LN827595_sLCL-IS1.03_sLCL-PTLD_Australia_ND | 7 | 0 | 7 | 0 | 3904 | 0 | 0 | 81602 |
| LN824204_HL05_HL_UK_ND | 15 | 0 | 15 | 0 | 15694 | 0 | 0 | 69812 |
| LC150338_HNNPC5_NPC_China_2013 | 116 | 110 | 6 | 3 | 49565 | 18.333334 | 0.5 | 45833 |
| LN827574_sLCL-1.09_sLCL-PTLD_Kenya_ND | 26 | 0 | 26 | 0 | 44323 | 0 | 0 | 41181 |
| KP968257_CCH_BL_Brazil_1980 | 45 | 21 | 24 | 1 | 28636 | 0.875 | 0.041667 | 61010 |
| LN827597_sLCL-IS1.04_sLCL-PTLD_Australia_ND | 20 | 0 | 20 | 0 | 0 | 0 | 0 | 85509 |
| KT273943_EBVaGC2_GC_China_2013 | 21 | 0 | 21 | 0 | 35550 | 0 | 0 | 49955 |
| KT273945_EBVaGC5_GC_China_2009 | 17 | 0 | 17 | 0 | 22551 | 0 | 0 | 62956 |
| LN824208_Akata-reseq_BL_Japan_ND | 1 | 0 | 1 | 0 | 35550 | 0 | 0 | 49955 |
| KT823508_LC3_LC_China_2014 | 19 | 9 | 10 | 1 | 45124 | 0.9 | 0.1 | 41809 |
| LN824142_Saliva1_Health_UK_ND | 158 | 60 | 98 | 2 | 28608 | 0.612245 | 0.020408 | 59411 |
| LN827551_Makau_BL_Kenya_ND | 0 | 0 | 0 | 0 | 20166 | 0 | 0 | 65337 |
| LN824206_pLCL-TRL-post_sLCL_USA_ND | 0 | 0 | 0 | 0 | 25736 | 0 | 0 | 59768 |
| KP968258_MP_BL_Brazil_1980 | 92 | 75 | 17 | 2 | 47810 | 4.411765 | 0.117647 | 53443 |
| KC207814_Mutu_BL_Kenya_ND | 12 | 0 | 12 | 0 | 4650 | 0 | 0 | 80855 |
| KT273947_EBVaGC7_GC_China_2009 | 19 | 0 | 19 | 0 | 35550 | 0 | 0 | 49955 |
| KP968260_VGO_BL_Brazil_1980 | 300 | 30 | 270 | 1 | 26390 | 0.111111 | 0.003704 | 61129 |
| KT273948_EBVaGC8_GC_China_2010 | 95 | 45 | 50 | 2 | 38437 | 0.9 | 0.04 | 55793 |
| KT273944_EBVaGC4_GC_China_2014 | 59 | 32 | 27 | 1 | 36031 | 1.185185 | 0.037037 | 49955 |
| AY961628_GD1_NPC-saliva_China_ND | 161 | 87 | 74 | 2 | 27296 | 1.175676 | 0.027027 | 64235 |
| KT823507_LC2_LC_China_2014 | 14 | 0 | 14 | 0 | 29128 | 0 | 0 | 56376 |
| LC149491_HNNPC2_NPC_China_2013 | 118 | 87 | 31 | 2 | 43215 | 2.806452 | 0.064516 | 45833 |
| KT254013_EBVaGC3_GC_China_2013 | 19 | 0 | 19 | 0 | 43693 | 0 | 0 | 41809 |
| KT273946_EBVaGC6_GC_China_2009 | 239 | 82 | 157 | 2 | 38345 | 0.522293 | 0.012739 | 49397 |
| KT823509_LC4_LC_China_2014 | 129 | 96 | 33 | 2 | 43618 | 2.909091 | 0.060606 | 48189 |
| LN827523_L591_Hl-cell-line_Germany_ND | 156 | 128 | 28 | 1 | 6940 | 4.571429 | 0.035714 | 81602 |
| LN824207_pLCL-TRL1-pre_sLCL_USA_ND | 0 | 0 | 0 | 0 | 25736 | 0 | 0 | 59768 |
| KT823506_LC1_LC_China_2014 | 17 | 0 | 17 | 0 | 35550 | 0 | 0 | 49955 |
| KF992565_HKNPC3_NPC-tumor_China_2008 | 4 | 0 | 4 | 0 | 39668 | 0 | 0 | 45833 |
| KF992568_HKNPC6_NPC-tumor_China_2008 | 19 | 0 | 19 | 0 | 30668 | 0 | 0 | 54837 |
| JQ009376_HKNPC1_NPC-tumor_China_2008 | 16 | 0 | 16 | 0 | 39668 | 0 | 0 | 45833 |
| LC150741_HNNPC6_NPC-tumor_China_2013 | 18 | 0 | 18 | 0 | 39668 | 0 | 0 | 45833 |
| LC150337_HNNPC4_NPC-tumor_China_2013 | 10 | 0 | 10 | 0 | 39668 | 0 | 0 | 45833 |
| KF992570_HKNPC8_NPC-tumor_China_2008 | 6 | 0 | 6 | 0 | 39668 | 0 | 0 | 45833 |
| LC137018_HNNPC1_NPC-tumor_China_2013 | 13 | 0 | 13 | 0 | 39668 | 0 | 0 | 45833 |
| KF992567_HKNPC5_NPC-tumor_China_2008 | 10 | 0 | 10 | 0 | 39668 | 0 | 0 | 45833 |
| KC207813_Akata_BL_Japan_ND | 1 | 0 | 1 | 0 | 35550 | 0 | 0 | 49955 |
| KC617875_C666-1_NPC-tumor_China_2011 | 4 | 0 | 4 | 0 | 39668 | 0 | 0 | 45829 |
| KF992566_HKNPC4_NPC-tumor_China_2008 | 10 | 0 | 10 | 0 | 39668 | 0 | 0 | 45833 |
| LC150742_HNNPC7_NPC-tumor_China_2013 | 83 | 54 | 29 | 2 | 29877 | 1.862069 | 0.068966 | 59138 |
| LC150327_HNNPC3_NPC-tumor_China_2013 | 14 | 0 | 14 | 0 | 39668 | 0 | 0 | 45828 |
| KJ411974_C666-1_NPC-tumor_China_ND | 0 | 0 | 0 | 0 | 39668 | 0 | 0 | 45833 |
| KF992571_HKNPC9_NPC-tumor_China_2008 | 8 | 0 | 8 | 0 | 39668 | 0 | 0 | 45833 |
| KP735248_GC1_GC-cell-line_SouthKorea_2010 | 4 | 0 | 4 | 0 | 36436 | 0 | 0 | 49071 |
| KF992569_HKNPC7_NPC-tumor_China_2008 | 7 | 0 | 7 | 0 | 30668 | 0 | 0 | 54837 |
| KX125052_SNU-719_GC-cell-line_SouthKorea_2008 | 0 | 0 | 0 | 0 | 36436 | 0 | 0 | 49071 |
| KF992564_HKNPC2_NPC-tumor_China_2008 | 43 | 40 | 3 | 1 | 40631 | 13.333333 | 0.333333 | 45833 |
| LC150743_HNNPC8_NPC-tumor_China_2013 | 28 | 0 | 28 | 0 | 39667 | 0 | 0 | 45828 |
| KX125050_GC-EBV1_GC_China_2011 | 73 | 61 | 12 | 1 | 40435 | 5.083333 | 0.083333 | 45833 |
| AP015015_SNU719_GC-cell-line_Korea_ND | 0 | 0 | 0 | 0 | 36436 | 0 | 0 | 49071 |
| KX125053_YCCEL1_GC_SouthKorea_2010 | 0 | 0 | 0 | 0 | 31626 | 0 | 0 | 53880 |
| KF717093_Raji_BL_Nigeria_ND | 280 | 126 | 154 | 4 | 30430 | 0.818182 | 0.025974 | 60428 |
| HQ020558_GD2_NPC-tumor_China_2009 | 19 | 0 | 19 | 0 | 39668 | 0 | 0 | 45833 |
| MF547453_E1583_BCv1_IM_USA_2013 | 3 | 0 | 3 | 0 | 3904 | 0 | 0 | 81602 |
| MF547454_E1583_OWv1_IM_USA_2013 | 42 | 0 | 42 | 0 | 3904 | 0 | 0 | 81594 |
| MF547455_E1583_BCv7_IM_USA_2013 | 96 | 0 | 96 | 0 | 0 | 0 | 0 | 85509 |
| MF547456_E1583_OWv7_IM_USA_2014 | 32 | 15 | 17 | 1 | 7922 | 0.882353 | 0.058824 | 81598 |
| MF547457_E1587_BCv1_IM_USA_2013 | 21 | 0 | 21 | 0 | 8486 | 0 | 0 | 77021 |
| MF547458_E1587_OWv1_IM_USA_2013 | 43 | 0 | 43 | 0 | 0 | 0 | 0 | 85505 |
| MF547460_E1587_OWv7_IM_USA_2014 | 41 | 0 | 41 | 0 | 0 | 0 | 0 | 85505 |
| MF547461_E1536_BCv1_IM_USA_2012 | 0 | 0 | 0 | 0 | 10340 | 0 | 0 | 75166 |
| MF547462_E1536_OWv1_IM_USA_2012 | 0 | 0 | 0 | 0 | 10340 | 0 | 0 | 75166 |
| MF547463_E1563_BCv1_IM_USA_2013 | 6 | 0 | 6 | 0 | 9952 | 0 | 0 | 75553 |
| MF547464_E1536_OWv7_IM_USA_2013 | 3 | 0 | 3 | 0 | 10340 | 0 | 0 | 75166 |
| MF547465_E1536_BCv7_IM_USA_2013 | 116 | 59 | 57 | 2 | 10600 | 1.035088 | 0.035088 | 83170 |
| MF547466_E1548_BCv1_IM_USA_2013 | 0 | 0 | 0 | 0 | 3722 | 0 | 0 | 81786 |
| MF547467_E1548_OWv1_IM_USA_2013 | 129 | 34 | 95 | 1 | 822 | 0.357895 | 0.010526 | 85501 |
| MF547468_E1548_BCv7_IM_USA_2013 | 5 | 0 | 5 | 0 | 0 | 0 | 0 | 85505 |
| MF547469_E1548_OWv7_IM_USA_2013 | 25 | 19 | 6 | 1 | 3980 | 3.166667 | 0.166667 | 81782 |
| MF547470_E1563_OWv1_IM_USA_2013 | 5 | 0 | 5 | 0 | 9952 | 0 | 0 | 75553 |
| MF547471_E1563_OWv7_IM_USA_2014 | 26 | 0 | 26 | 0 | 9952 | 0 | 0 | 75549 |
| MF547472_E1563_BCV7_IM_USA_2014 | 152 | 0 | 152 | 0 | 0 | 0 | 0 | 85496 |
| MF547473_E1590_BCv1_IM_USA_2014 | 2 | 0 | 2 | 0 | 8486 | 0 | 0 | 77021 |
| MF547474_E1590_OWv1_IM_USA_2014 | 23 | 0 | 23 | 0 | 8486 | 0 | 0 | 77017 |
| MF547475_E1590_BCv7_IM_USA_2014 | 57 | 0 | 57 | 0 | 0 | 0 | 0 | 85498 |
| MF547476_E1590_OWv7_IM_USA_2014 | 59 | 0 | 59 | 0 | 0 | 0 | 0 | 85488 |
| MF547477_E1492_BCv1_IM_USA_2010 | 0 | 0 | 0 | 0 | 2393 | 0 | 0 | 83113 |
| MF547478_E1492_OWv1_IM_USA_2010 | 0 | 0 | 0 | 0 | 2393 | 0 | 0 | 83113 |
| MF547479_E1492_BCv7_IM_USA_2011 | 37 | 20 | 17 | 1 | 2934 | 1.176471 | 0.058824 | 83427 |
| MF547480_E1492_OWv7_IM_USA_2011 | 62 | 0 | 62 | 0 | 1094 | 0 | 0 | 84411 |
| MF547481_E1503_BCv1_IM_USA_2010 | 0 | 0 | 0 | 0 | 2079 | 0 | 0 | 83428 |
| MF547482_E1503_OWv1_IM_USA_2010 | 0 | 0 | 0 | 0 | 2079 | 0 | 0 | 83428 |
| MF547483_E1503_BCv7_IM_USA_2011 | 8 | 0 | 8 | 0 | 2079 | 0 | 0 | 83428 |
| MF547484_E1503_OWv7_IM_USA_2011 | 2 | 0 | 2 | 0 | 2079 | 0 | 0 | 83428 |
| MF547485_E1578_BCv1_IM_USA_2013 | 74 | 0 | 74 | 0 | 14707 | 0 | 0 | 70791 |
| MF547486_E1578_BCv7_IM_USA_2014 | 227 | 24 | 203 | 1 | 1423 | 0.118227 | 0.004926 | 85502 |
| MF547487_E1578_OWv7_IM_USA_2014 | 30 | 0 | 30 | 0 | 0 | 0 | 0 | 85501 |
| MF547488_E1578_OWv1_IM_USA_2013 | 69 | 22 | 47 | 1 | 15658 | 0.468085 | 0.021277 | 70799 |
| MF547489_E1577_BCv1_IM_USA_2013 | 0 | 0 | 0 | 0 | 21767 | 0 | 0 | 63736 |
| MF547490_E1577_OWv1_IM_USA_2013 | 0 | 0 | 0 | 0 | 21767 | 0 | 0 | 63736 |
| MF547491_E1577_OWv7_IM_USA_ND | 27 | 0 | 27 | 0 | 21767 | 0 | 0 | 63735 |
| MF547492_E1577_BCv7_IM_USA_ND | 170 | 0 | 170 | 0 | 0 | 0 | 0 | 85498 |
| MG021314_EBVaGC8-2_GC_China_2014 | 9 | 0 | 9 | 0 | 41073 | 0 | 0 | 44432 |
| MG021305_YCCEL1-GC1_GC_China_2014 | 16 | 0 | 16 | 0 | 31626 | 0 | 0 | 53880 |
| MG021315_EBVaGC8-3_GC_China_2014 | 0 | 0 | 0 | 0 | 41073 | 0 | 0 | 44432 |
| MG021317_EBVaGC5-1_GC_USA_2013 | 117 | 82 | 35 | 2 | 23246 | 2.342857 | 0.057143 | 64299 |
| MG021308_Mutu-GC1_GC_USA_2013 | 63 | 0 | 63 | 0 | 3904 | 0 | 0 | 81602 |
| MG021307_Akata-GC1_GC_USA_2013 | 80 | 23 | 57 | 1 | 30119 | 0.403509 | 0.017544 | 55387 |
| MG021312_Akata-GC1_GC_USA_2017 | 28 | 16 | 12 | 1 | 23133 | 1.333333 | 0.083333 | 64916 |
| MG021316_HKNPC6-GC1_GC_Vietnam_2013 | 388 | 48 | 340 | 2 | 15069 | 0.141176 | 0.005882 | 70799 |
| MG021310_Mutu-GC3_GC_USA_2013 | 22 | 12 | 10 | 1 | 11078 | 1.2 | 0.1 | 74428 |
| MG021311_Mutu-GC4_GC_USA_2013 | 23 | 12 | 11 | 1 | 11078 | 1.090909 | 0.090909 | 74428 |
| MG021309_Mutu-GC2_GC_USA_2013 | 154 | 94 | 60 | 3 | 31166 | 1.566667 | 0.05 | 54800 |
| MG021313_EBVaGC8-1_GC_USA_2013 | 30 | 0 | 30 | 0 | 41073 | 0 | 0 | 44432 |
| MG021306_YCCEL1-GC2_GC_Poland_2013 | 20 | 0 | 20 | 0 | 31626 | 0 | 0 | 53880 |
| N8 | 48 | 0 | 48 | 0 | 3722 | 0 | 0 | 81786 |
| N7 | 27 | 12 | 15 | 1 | 3722 | 0.8 | 0.066667 | 83171 |
| N6 | 15 | 9 | 6 | 1 | 2337 | 1.5 | 0.166667 | 85509 |
| N5 | 14 | 0 | 14 | 0 | 0 | 0 | 0 | 85509 |
| N15 | 32 | 0 | 32 | 0 | 0 | 0 | 0 | 85505 |
| N14 | 21 | 0 | 21 | 0 | 0 | 0 | 0 | 85508 |
| N25 | 18 | 0 | 18 | 0 | 21767 | 0 | 0 | 63736 |
| N24 | 55 | 37 | 18 | 2 | 21767 | 2.055556 | 0.111111 | 67154 |
| N23 | 181 | 72 | 109 | 2 | 18351 | 0.66055 | 0.018349 | 77021 |
| N30 | 3 | 0 | 3 | 0 | 8486 | 0 | 0 | 77021 |
| N29 | 4 | 0 | 4 | 0 | 8486 | 0 | 0 | 77021 |
| N36 | 82 | 79 | 3 | 1 | 11078 | 26.333334 | 0.333333 | 77021 |
| N35 | 0 | 0 | 0 | 0 | 8486 | 0 | 0 | 77021 |
| N34 | 0 | 0 | 0 | 0 | 8486 | 0 | 0 | 77021 |
| N33 | 0 | 0 | 0 | 0 | 8486 | 0 | 0 | 77021 |
| N37 | 8 | 0 | 8 | 0 | 8486 | 0 | 0 | 77021 |
| N32 | 5 | 0 | 5 | 0 | 8486 | 0 | 0 | 77021 |
| N31 | 1 | 0 | 1 | 0 | 8486 | 0 | 0 | 77021 |
| N28 | 9 | 0 | 9 | 0 | 8486 | 0 | 0 | 77021 |
| N27 | 24 | 0 | 24 | 0 | 8486 | 0 | 0 | 77021 |
| N26 | 45 | 0 | 45 | 0 | 8486 | 0 | 0 | 77021 |
| N22 | 101 | 28 | 73 | 1 | 8486 | 0.383562 | 0.013699 | 84414 |
| N42 | 1 | 0 | 1 | 0 | 10413 | 0 | 0 | 75094 |
| N41 | 230 | 0 | 230 | 0 | 10413 | 0 | 0 | 75094 |
| N43 | 34 | 0 | 34 | 0 | 10413 | 0 | 0 | 75094 |
| N40 | 604 | 517 | 87 | 1 | 10413 | 5.942529 | 0.011494 | 76245 |
| N50 | 40 | 30 | 10 | 1 | 20039 | 3 | 0.1 | 69812 |
| N49 | 0 | 0 | 0 | 0 | 15694 | 0 | 0 | 69812 |
| N51 | 0 | 0 | 0 | 0 | 15694 | 0 | 0 | 69812 |
| N48 | 1 | 0 | 1 | 0 | 15694 | 0 | 0 | 69812 |
| N47 | 229 | 79 | 150 | 2 | 15694 | 0.526667 | 0.013333 | 70799 |
| N54 | 74 | 0 | 74 | 0 | 14707 | 0 | 0 | 70799 |
| N53 | 124 | 45 | 79 | 1 | 14707 | 0.56962 | 0.012658 | 70799 |
| N56 | 64 | 0 | 64 | 0 | 14707 | 0 | 0 | 70799 |
| N63 | 0 | 0 | 0 | 0 | 36436 | 0 | 0 | 49071 |
| N62 | 251 | 222 | 29 | 2 | 36436 | 7.655172 | 0.068966 | 62956 |
| N61 | 0 | 0 | 0 | 0 | 22551 | 0 | 0 | 62956 |
| N65 | 3 | 0 | 3 | 0 | 31626 | 0 | 0 | 53880 |
| N68 | 0 | 0 | 0 | 0 | 31626 | 0 | 0 | 53880 |
| N67 | 0 | 0 | 0 | 0 | 31626 | 0 | 0 | 53880 |
| N66 | 19 | 0 | 19 | 0 | 31626 | 0 | 0 | 53880 |
| N64 | 28 | 27 | 1 | 1 | 31626 | 27 | 1 | 62956 |
| N60 | 27 | 21 | 6 | 1 | 22551 | 3.5 | 0.166667 | 64299 |
| N59 | 20 | 0 | 20 | 0 | 21208 | 0 | 0 | 64299 |
| N58 | 81 | 41 | 40 | 1 | 21208 | 1.025 | 0.025 | 66870 |
| N74 | 26 | 0 | 26 | 0 | 41073 | 0 | 0 | 44432 |
| N73 | 67 | 57 | 10 | 2 | 41073 | 5.7 | 0.2 | 55793 |
| N72 | 77 | 74 | 3 | 3 | 29712 | 24.666666 | 1 | 63713 |
| N79 | 0 | 0 | 0 | 0 | 35550 | 0 | 0 | 49955 |
| N80 | 0 | 0 | 0 | 0 | 35550 | 0 | 0 | 49955 |
| N78 | 0 | 0 | 0 | 0 | 35550 | 0 | 0 | 49955 |
| N83 | 40 | 33 | 7 | 1 | 43693 | 4.714286 | 0.142857 | 48189 |
| N82 | 25 | 22 | 3 | 2 | 37314 | 7.333333 | 0.666667 | 49955 |
| N81 | 0 | 0 | 0 | 0 | 35550 | 0 | 0 | 49955 |
| N77 | 0 | 0 | 0 | 0 | 35550 | 0 | 0 | 49955 |
| N84 | 22 | 0 | 22 | 0 | 35550 | 0 | 0 | 49955 |
| N76 | 65 | 37 | 28 | 1 | 35550 | 1.321429 | 0.035714 | 55387 |
| N75 | 72 | 60 | 12 | 2 | 30119 | 5 | 0.166667 | 63713 |
| N71 | 95 | 75 | 20 | 1 | 21792 | 3.75 | 0.05 | 64235 |
| N70 | 30 | 14 | 16 | 1 | 21270 | 0.875 | 0.0625 | 65527 |
| N91 | 18 | 10 | 8 | 1 | 39668 | 1.25 | 0.125 | 45833 |
| N95 | 0 | 0 | 0 | 0 | 39668 | 0 | 0 | 45833 |
| N94 | 16 | 0 | 16 | 0 | 39668 | 0 | 0 | 45833 |
| N93 | 0 | 0 | 0 | 0 | 39668 | 0 | 0 | 45833 |
| N102 | 4 | 0 | 4 | 0 | 39668 | 0 | 0 | 45833 |
| N101 | 0 | 0 | 0 | 0 | 39668 | 0 | 0 | 45833 |
| N103 | 0 | 0 | 0 | 0 | 39668 | 0 | 0 | 45833 |
| N100 | 0 | 0 | 0 | 0 | 39668 | 0 | 0 | 45833 |
| N104 | 1 | 0 | 1 | 0 | 39668 | 0 | 0 | 45833 |
| N99 | 0 | 0 | 0 | 0 | 39668 | 0 | 0 | 45833 |
| N98 | 2 | 0 | 2 | 0 | 39668 | 0 | 0 | 45833 |
| N97 | 0 | 0 | 0 | 0 | 39668 | 0 | 0 | 45833 |
| N108 | 0 | 0 | 0 | 0 | 39668 | 0 | 0 | 45833 |
| N107 | 5 | 0 | 5 | 0 | 39668 | 0 | 0 | 45833 |
| N106 | 0 | 0 | 0 | 0 | 39668 | 0 | 0 | 45833 |
| N105 | 1 | 0 | 1 | 0 | 39668 | 0 | 0 | 45833 |
| N96 | 0 | 0 | 0 | 0 | 39668 | 0 | 0 | 45833 |
| N92 | 0 | 0 | 0 | 0 | 39668 | 0 | 0 | 45833 |
| N90 | 86 | 54 | 32 | 3 | 39668 | 1.6875 | 0.09375 | 49397 |
| N89 | 179 | 148 | 31 | 2 | 36106 | 4.774194 | 0.064516 | 59411 |
| N111 | 52 | 47 | 5 | 2 | 30668 | 9.4 | 0.4 | 59411 |
| N112 | 156 | 134 | 22 | 3 | 39412 | 6.090909 | 0.136364 | 59411 |
| N110 | 13 | 0 | 13 | 0 | 26093 | 0 | 0 | 59411 |
| N114 | 33 | 31 | 2 | 1 | 29128 | 15.5 | 0.5 | 59138 |
| N113 | 49 | 38 | 11 | 1 | 26366 | 3.454545 | 0.090909 | 59411 |
| N109 | 72 | 0 | 72 | 0 | 26093 | 0 | 0 | 59411 |
| N88 | 100 | 0 | 100 | 0 | 26093 | 0 | 0 | 59411 |
| N87 | 44 | 16 | 28 | 1 | 26093 | 0.571429 | 0.035714 | 61129 |
| N86 | 36 | 0 | 36 | 0 | 24376 | 0 | 0 | 61129 |
| N85 | 52 | 34 | 18 | 2 | 24376 | 1.888889 | 0.111111 | 65527 |
| N69 | 34 | 14 | 20 | 1 | 19979 | 0.7 | 0.05 | 66870 |
| N57 | 127 | 52 | 75 | 2 | 18636 | 0.693333 | 0.026667 | 70799 |
| N55 | 18 | 0 | 18 | 0 | 14707 | 0 | 0 | 70799 |
| N52 | 69 | 24 | 45 | 1 | 14707 | 0.533333 | 0.022222 | 70799 |
| N46 | 57 | 0 | 57 | 0 | 14707 | 0 | 0 | 70799 |
| N115 | 37 | 0 | 37 | 0 | 14707 | 0 | 0 | 70799 |
| N45 | 43 | 0 | 43 | 0 | 14707 | 0 | 0 | 70799 |
| N44 | 87 | 37 | 50 | 1 | 14707 | 0.74 | 0.02 | 76245 |
| N39 | 97 | 50 | 47 | 1 | 9262 | 1.06383 | 0.021277 | 84414 |
| N124 | 0 | 0 | 0 | 0 | 4650 | 0 | 0 | 80855 |
| N123 | 0 | 0 | 0 | 0 | 4650 | 0 | 0 | 80855 |
| N122 | 4 | 0 | 4 | 0 | 4650 | 0 | 0 | 80855 |
| N126 | 0 | 0 | 0 | 0 | 4650 | 0 | 0 | 80855 |
| N125 | 6 | 0 | 6 | 0 | 4650 | 0 | 0 | 80855 |
| N121 | 0 | 0 | 0 | 0 | 4650 | 0 | 0 | 80855 |
| N127 | 8 | 0 | 8 | 0 | 4650 | 0 | 0 | 80855 |
| N120 | 0 | 0 | 0 | 0 | 4650 | 0 | 0 | 80855 |
| N119 | 142 | 0 | 142 | 0 | 4650 | 0 | 0 | 80855 |
| N118 | 83 | 12 | 71 | 1 | 4650 | 0.169014 | 0.014085 | 81028 |
| N128 | 64 | 0 | 64 | 0 | 4478 | 0 | 0 | 81028 |
| N117 | 38 | 17 | 21 | 1 | 4478 | 0.809524 | 0.047619 | 83295 |
| N116 | 34 | 10 | 24 | 1 | 2212 | 0.416667 | 0.041667 | 84414 |
| N38 | 22 | 0 | 22 | 0 | 1094 | 0 | 0 | 84414 |
| N21 | 36 | 0 | 36 | 0 | 1094 | 0 | 0 | 84414 |
| N134 | 3 | 0 | 3 | 0 | 20589 | 0 | 0 | 64916 |
| N136 | 0 | 0 | 0 | 0 | 20589 | 0 | 0 | 64916 |
| N135 | 0 | 0 | 0 | 0 | 20589 | 0 | 0 | 64916 |
| N133 | 748 | 579 | 169 | 3 | 20589 | 3.426035 | 0.017751 | 66909 |
| N144 | 0 | 0 | 0 | 0 | 44323 | 0 | 0 | 41181 |
| N143 | 12 | 9 | 3 | 1 | 44323 | 3 | 0.333333 | 41181 |
| N142 | 4 | 0 | 4 | 0 | 44323 | 0 | 0 | 41181 |
| N141 | 0 | 0 | 0 | 0 | 44323 | 0 | 0 | 41181 |
| N145 | 1 | 0 | 1 | 0 | 44323 | 0 | 0 | 41181 |
| N140 | 121 | 114 | 7 | 4 | 44323 | 16.285715 | 0.571429 | 61010 |
| N139 | 104 | 31 | 73 | 1 | 24496 | 0.424658 | 0.013699 | 66909 |
| N147 | 167 | 71 | 96 | 2 | 25736 | 0.739583 | 0.020833 | 66909 |
| N148 | 611 | 568 | 43 | 3 | 24630 | 13.209302 | 0.069767 | 66909 |
| N146 | 10 | 0 | 10 | 0 | 18596 | 0 | 0 | 66909 |
| N138 | 21 | 0 | 21 | 0 | 18596 | 0 | 0 | 66909 |
| N153 | 137 | 66 | 71 | 1 | 32060 | 0.929577 | 0.014085 | 60428 |
| N152 | 51 | 0 | 51 | 0 | 25075 | 0 | 0 | 60428 |
| N154 | 146 | 43 | 103 | 1 | 26190 | 0.417476 | 0.009709 | 60428 |
| N151 | 16 | 0 | 16 | 0 | 25075 | 0 | 0 | 60428 |
| N150 | 28 | 0 | 28 | 0 | 25075 | 0 | 0 | 60428 |
| N155 | 80 | 64 | 16 | 2 | 30703 | 4 | 0.125 | 60428 |
| N149 | 31 | 30 | 1 | 2 | 25075 | 30 | 2 | 66909 |
| N137 | 15 | 0 | 15 | 0 | 18596 | 0 | 0 | 66909 |
| N132 | 48 | 34 | 14 | 1 | 18596 | 2.428571 | 0.071429 | 74584 |
| N156 | 133 | 72 | 61 | 2 | 24360 | 1.180328 | 0.032787 | 74584 |
| N131 | 25 | 0 | 25 | 0 | 10922 | 0 | 0 | 74584 |
| N158 | 351 | 129 | 222 | 4 | 20166 | 0.581081 | 0.018018 | 69283 |
| N157 | 156 | 82 | 74 | 1 | 16222 | 1.108108 | 0.013514 | 74584 |
| N130 | 128 | 85 | 43 | 3 | 10922 | 1.976744 | 0.069767 | 84414 |
| N160 | 30 | 20 | 10 | 1 | 9679 | 2 | 0.1 | 84414 |
| N166 | 8 | 0 | 8 | 0 | 3904 | 0 | 0 | 81602 |
| N165 | 1 | 0 | 1 | 0 | 3904 | 0 | 0 | 81602 |
| N169 | 9 | 0 | 9 | 0 | 3904 | 0 | 0 | 81602 |
| N168 | 14 | 0 | 14 | 0 | 3904 | 0 | 0 | 81602 |
| N167 | 0 | 0 | 0 | 0 | 3904 | 0 | 0 | 81602 |
| N164 | 2 | 0 | 2 | 0 | 3904 | 0 | 0 | 81602 |
| N173 | 15 | 0 | 15 | 0 | 9952 | 0 | 0 | 75553 |
| N172 | 114 | 36 | 78 | 1 | 9952 | 0.461538 | 0.012821 | 81602 |
| N171 | 4 | 0 | 4 | 0 | 3904 | 0 | 0 | 81602 |
| N170 | 0 | 0 | 0 | 0 | 3904 | 0 | 0 | 81602 |
| N163 | 18 | 11 | 7 | 1 | 3904 | 1.571429 | 0.142857 | 83428 |
| N175 | 14 | 8 | 6 | 1 | 2393 | 1.333333 | 0.166667 | 83428 |
| N174 | 35 | 0 | 35 | 0 | 2079 | 0 | 0 | 83428 |
| N162 | 1 | 0 | 1 | 0 | 2079 | 0 | 0 | 83428 |
| N179 | 0 | 0 | 0 | 0 | 10640 | 0 | 0 | 74867 |
| N178 | 37 | 30 | 7 | 1 | 10640 | 4.285714 | 0.142857 | 80734 |
| N181 | 1 | 0 | 1 | 0 | 10340 | 0 | 0 | 75166 |
| N180 | 78 | 54 | 24 | 2 | 10340 | 2.25 | 0.083333 | 80734 |
| N177 | 70 | 47 | 23 | 1 | 4773 | 2.043478 | 0.043478 | 83428 |
| N185 | 0 | 0 | 0 | 0 | 2079 | 0 | 0 | 83428 |
| N184 | 2 | 0 | 2 | 0 | 2079 | 0 | 0 | 83428 |
| N183 | 33 | 0 | 33 | 0 | 2079 | 0 | 0 | 83428 |
| N182 | 9 | 0 | 9 | 0 | 2079 | 0 | 0 | 83428 |
| N176 | 2 | 0 | 2 | 0 | 2079 | 0 | 0 | 83428 |
| N161 | 63 | 18 | 45 | 1 | 2079 | 0.4 | 0.022222 | 84414 |
| N159 | 22 | 0 | 22 | 0 | 1094 | 0 | 0 | 84414 |
| N129 | 9 | 0 | 9 | 0 | 1094 | 0 | 0 | 84414 |
| N20 | 29 | 0 | 29 | 0 | 1094 | 0 | 0 | 84414 |
| N19 | 21 | 8 | 13 | 1 | 1094 | 0.615385 | 0.076923 | 85509 |
| N18 | 42 | 0 | 42 | 0 | 0 | 0 | 0 | 85509 |
| N17 | 33 | 0 | 33 | 0 | 0 | 0 | 0 | 85509 |
| N16 | 12 | 0 | 12 | 0 | 0 | 0 | 0 | 85509 |
| N13 | 13 | 0 | 13 | 0 | 0 | 0 | 0 | 85509 |
| N12 | 24 | 0 | 24 | 0 | 0 | 0 | 0 | 85509 |
| N11 | 17 | 0 | 17 | 0 | 0 | 0 | 0 | 85509 |
| N186 | 30 | 0 | 30 | 0 | 0 | 0 | 0 | 85508 |
| N10 | 12 | 0 | 12 | 0 | 0 | 0 | 0 | 85509 |
| N9 | 3 | 0 | 3 | 0 | 0 | 0 | 0 | 85509 |
| N4 | 8 | 0 | 8 | 0 | 0 | 0 | 0 | 85509 |
| N3 | 0 | 0 | 0 | 0 | 0 | 0 | 0 | 85509 |
| N2 | 0 | 0 | 0 | 0 | 0 | 0 | 0 | 85509 |
| N1 | 0 | 0 | 0 | 0 | 0 | 0 | 0 | 85509 |

**
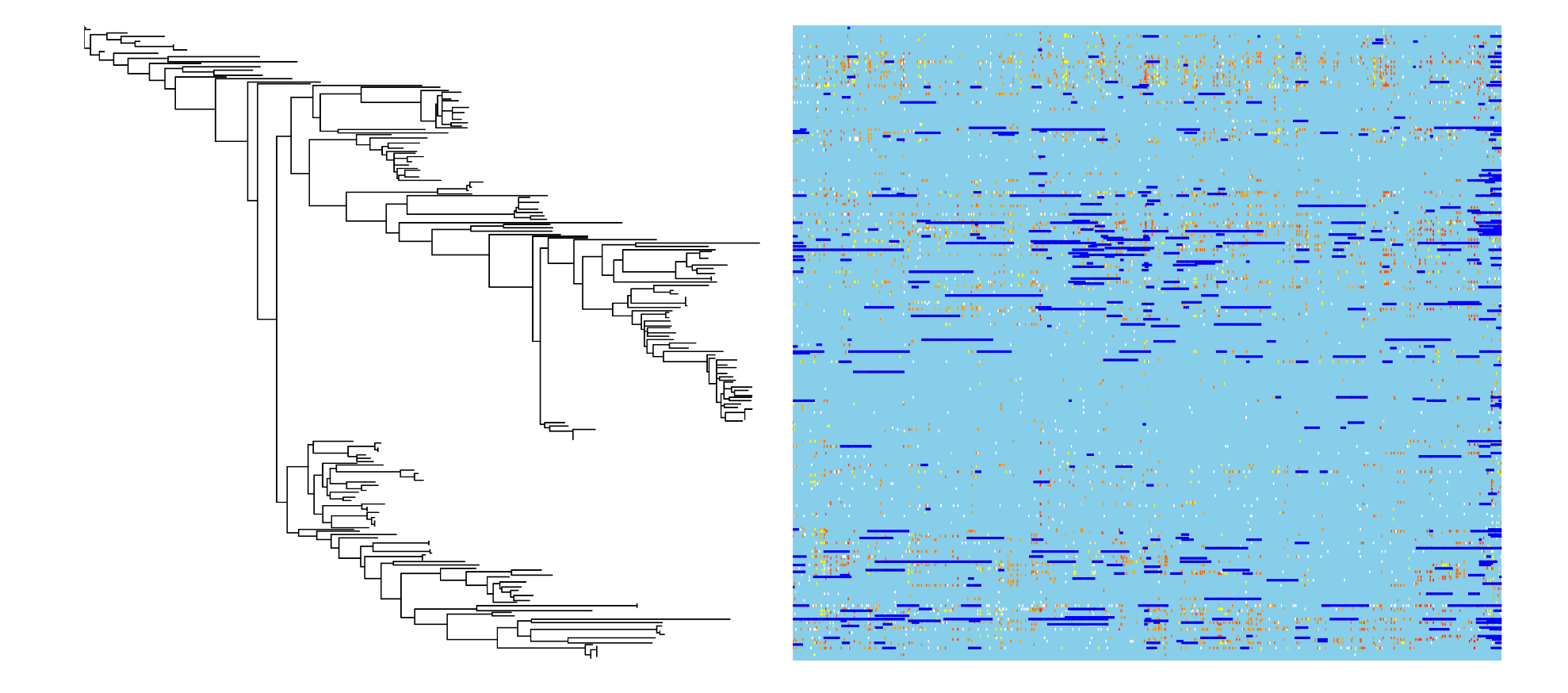
**

**Supplementary Figure S1** The phylogenetic tree and putative recombination sites within the EBV genome. The graph shows a non-uniform distribution of recombination signals along the genome. Dark blue horizontal bars indicate recombination events detected in the genome, light blue vertical sites refer to areas without substitution, the white dots represent areas with non-homoplastic substitutions and the other color represents homoplastic substitutions. The recombination areas were highly frequent in the EBV genome with a certain tendency to be present in specific clades.


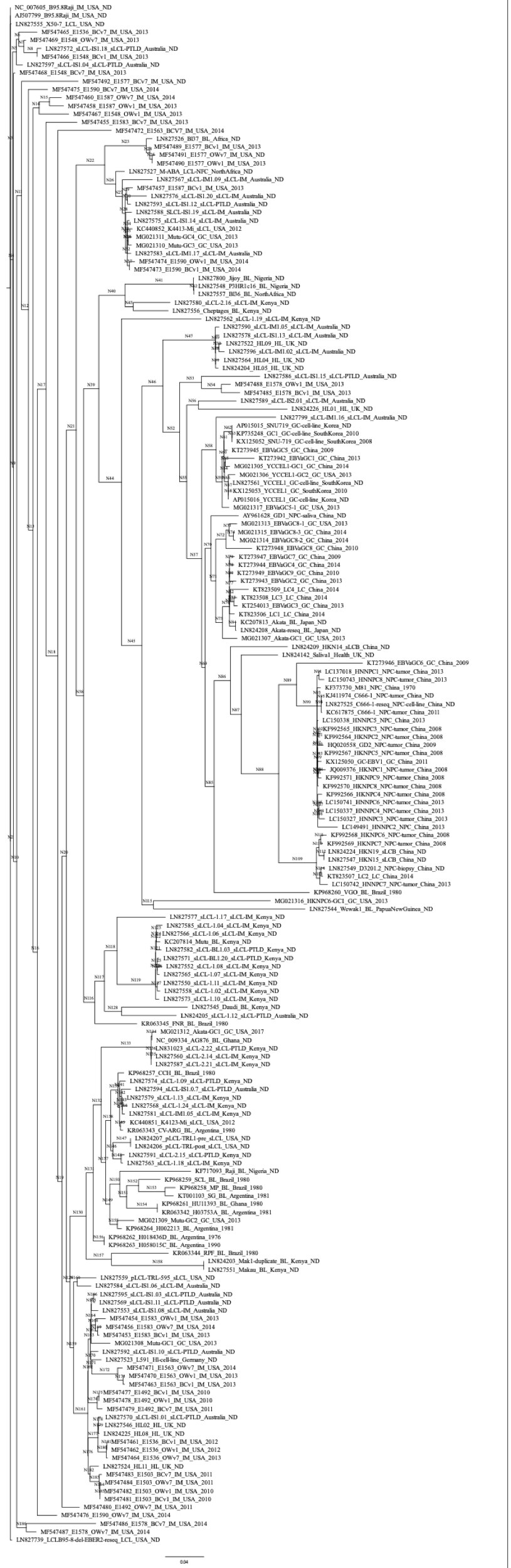


**Supplementary Figure S2** Phylogenetic tree based in genome information masked by Gubbings. The internal numbers indicate the node number.


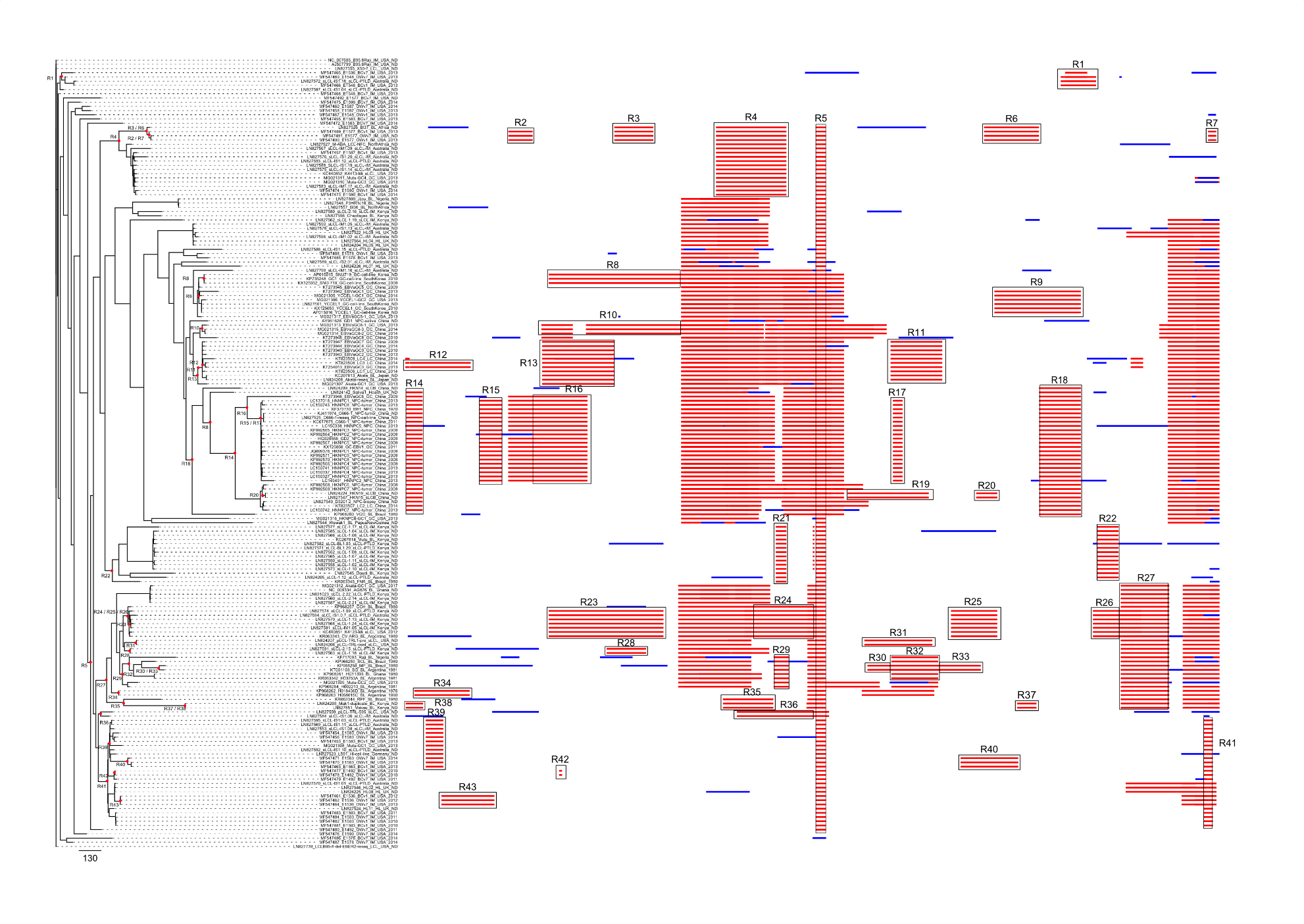


**Supplementary Figure S3**. Recombination events in the EBV genome according to phylogenetic clusters. The phylogenetic tree of the EBV genome and the plot representing the recombination along the genome. The recombination regions present in multiple strains are plotted in red lines, while those present in single strains are plotted in blue. The numerated black squares show that the recombination regions coincide with the phylogenetic clades. The most recombinant regions identified within a group of isolates were possibly acquired from a common ancestor.


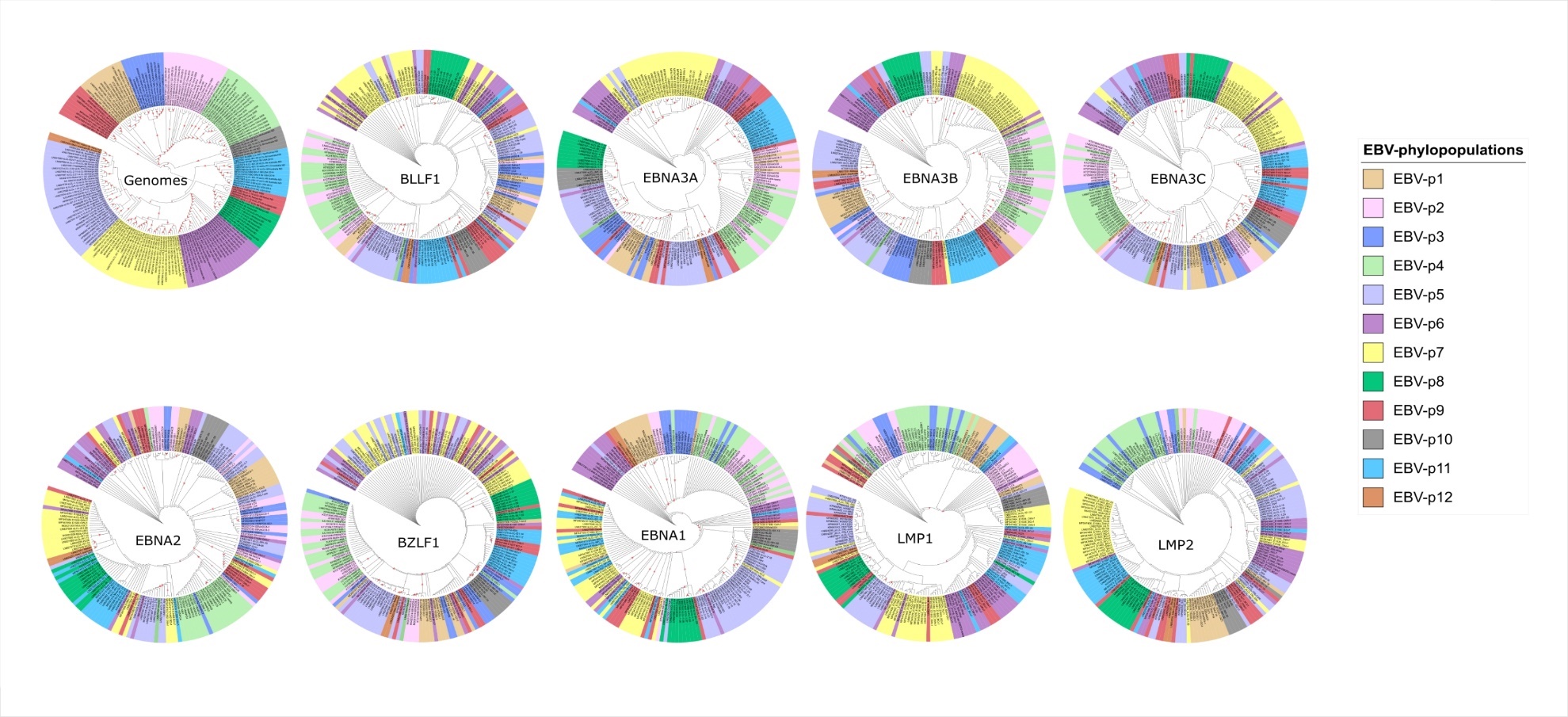


**Supplementary Figure S4.** Overlap between population and phylogenetic groups of ML trees based on sequences of whole genome (~85000pb), BLLF1 (2228pb), EBNA3A (2659pb), EBNA3B (2286pb), EBNA3C (2577pb), EBNA2 (1095pb), BZLF1 (801pb), EBNA1 (1019pb), LMP1 (1088pb) and LMP2 (1418pb). Branches are colored according to the 12 EBV-phylopopulation identified. The red triangles indicate bootstrap values over 80%.
